# *Alpiniae oxyphyllae fructus* improves production performance and egg quality of laying breeder hens by regulating reproductive hormones, antioxidant function, immunity and intestinal health

**DOI:** 10.1016/j.psj.2024.103770

**Published:** 2024-04-15

**Authors:** Mengjie Liu, Jianchi Lun, Gengxiong Huang, Yongqi Zhu, Wenbo Zhang, Wenxin Jin, Yiqing Ding, Shilong Liu, Qian Qu, Weijie Lv, Shining Guo

**Affiliations:** ⁎ College of Veterinary Medicine, South China Agricultural University, Guangzhou, PR China; † Guangdong Technology Research center for Traditional Chinese Veterinary Medicine and Natural Medicine, Guangzhou, PR China; ‡ International Institute of Traditional Chinese Veterinary Medicine, Guangzhou, PR China

**Keywords:** alpiniae oxyphyllae fructus, production performance, egg quality, immunity, intestinal health

## Abstract

*Alpiniae oxyphylla fructus* was extensively utilized both as dietary supplements and traditional herbal medicines for healthcare functions and has exhibited a positive impact on animal health. The present study aimed to investigate the effects of *Alpiniae oxyphyllae fructus* powder (**AOP**) on production performance, egg quality, egg yolk fatty acid composition, reproductive hormones, antioxidant capacity, immunity, anti-apoptosis ability, and intestinal health in hens. A total of 252 Hainan Wenchang laying hens (30-wk-old) were randomly divided into 3 groups with 6 replicates, a basic diet with 0 (**CON**), 1 g/kg AOP (**AOP1**), and 3 g/kg (**AOP3**) mixed AOP. The AOP supplementation was found to decrease the feed conversion ratio and embryo mortality but to increase the laying rate, average egg weight, and oviduct index linearly (*p* < 0.05). Furthermore, AOP treatment reduced the total saturated fatty acids and palmitic acid (C16:0) in the egg yolk while increasing eggshell strength, albumen height, and Haugh unit (*p* < 0.05). The serum levels of albumin and phosphorus were increased, whereas total cholesterol, triglycerides, and glucose levels decreased as a result of AOP treatment (*p* < 0.05). The inclusion of 3 g/kg AOP had higher 17 β-estradiol and follicle-stimulating hormone levels in serum, while it up-regulated follicle-stimulating hormone receptor and gonadotropin-releasing hormone expression in ovary (*p* < 0.05). Dietary AOP strengthened the expression of nuclear factor erythroid2-related factor 2 in ovary and increased the activity of superoxide dismutase and total antioxidant capacity, but had a lower malondialdehyde content in serum (*p* < 0.05). AOP at 3 g/kg up-regulated superoxide dismutase 1 and heme oxygenase 1 expression in jejunum and ovary (*p* < 0.05). Meanwhile, AOP supplementation down-regulated p53 expression in ovary and bcl-2-associated x expression in liver and jejunum, especially 3 g/kg of AOP had lower caspase-8 concentrations and down-regulated bcl-2-associated x and caspase-3 expression in ovary (*p* < 0.05). AOP treatment increased serum levels of immunoglobulin A and immunoglobulin M and upregulated interleukin-4 expression in the liver, while decreasing interleukin-1β expression in liver and ovary and nod-like receptor protein 3 expression in jejunum (*p* < 0.05). Dietary AOP increased the ratio of villus height to crypt depth but decreased crypt depth in jejunum, especially when 1 g/kg AOP increased expression levels of occludin, mucin-2, peptide-transporter 1, and sodium glucose cotransporter 1 in jejunum (*p* < 0.05). AOP treatment altered the composition of the cecal microbial community, as evidenced by increased abundance of *Oscillospira* and *Phascolarctobacterium* and reduced richness of *Clostridiaceae_Clostridium*. Dietary AOP supplementation enriched lipid, amino acid, and propanoate metabolism. Spearman's correlation analysis revealed that the genera *Oscillospira, Blautia*, and *Megasphaera* were related to laying performance and intestinal integrity. In brief, supplementation of AOP, especially at 3 g/kg, could improve production performance and egg quality of hens via modulating reproductive hormones, antioxidant capacity, immunity, intestinal barrier, and cecal microbiota. Overall, the present work recommends the dietary inclusion of AOP as a beneficial additive for improving the performance of hens.

## INTRODUCTION

The extensive use of antibiotics in chicken farming raised the risk of antibiotic residues in livestock products and fosters the growth of drug-resistant bacteria, which was extremely dangerous for human health and the environment (Al-Khalaifa et al., 2019). Wenchang chickens, which originated from Hainan island in the South China Sea, were known for their excellent meat quality and high prolificacy (Tang et al., 2009). Due to restrictions on antibiotic growth promoters for animals, intensive breeding of laying hens faced significant challenges, including reduced efficiency in production and impaired reproductive system function (Wang et al., 2018), a risk for intestinal barrier dysfunction, immune imbalance, and gut microbiota disturbance (Feng et al., 2021). Based on these facts, the search for safe and effective antibiotic alternatives or feed additives like medicinal plants to mitigated the impact of detrimental factors and ensure animal health was becoming increasingly urgent, especially in the context of the highly efficient production of hens.

Dried fruit from *Alpinia oxyphylla Miq*., commonly known as *A. oxyphylla*, was a dietetic Chinese herb (*Zingiberaceae*) that was extensively used in southern China to treat kidney asthenia, dyspepsia, diarrhea, and abdominal pain (Li et al., 2021). *Alpiniae oxyphyllae fructus* contained a variety of natural products, including flavonoids, essential oils, sesquiterpenes, diarylheptanoids, steroids, and glycosides (Chen et al., 2013; Zhang et al., 2018). Research has indicated that the primary active ingredients in *A. oxyphylla* include tectochrysin, nootkatone, chrysin, and protocatechuic acid (Xiao et al., 2023). Recent pharmacological studies have indicated that *A. oxyphylla s* exhibits a variety of biological effects, including anti-inflammatory (Wang et al., 2018), anti-apoptotic (Qi et al., 2019), antioxidant (El-Sonbaty et al., 2019), and anti-ulcer (Wang et al., 2012) properties. Procyanidin B-2 and epicatechin, the 2 primary chemical constituents of *A. oxyphylla*, have demonstrated 2,2-diphenyl-1-picryhydrazyl radical (**DPPH**) and 2,20 -azino-bis (3-ethylbenzo-thiazoline-6-sulfonic acid radical (**ABTS**) radical scavenging properties (Li et al., 2023). Additionally, a number of studies revealed that by enhancing antioxidant status, oregano essential oil reduced the detrimental effects of transportation on pigs (Zou et al., 2016). Earlier studies have demonstrated that flavonoids offer various beneficial effects, including enhancing immunity and improving gut morphology and function (Ali, 2015). One of the many natural compounds known as polyphenols regulated immune responses in the intestinal mucosa and enhanced protection against foreign infections through various mechanisms (Ding et al., 2018). The gut microbiome was essential for preserving gut health and had an effect on laying hens' general performance. In addition to providing vital micronutrients, amino acids, and short-chain fatty acids, it also aided in the intestinal epithelium's maturation and boosted the immune system's development by generating antimicrobial compounds and using competitive exclusion to stop pathogen invasion (Khan et al., 2020). Research has indicated that supplementation of ducks' diets with extracts from *A. oxyphylla* altered the intestinal microbial composition while preserving intestinal integrity (Ji et al., 2021). Furthermore, it has been discovered that flavonoids alter the gut microbiome's composition, inhibit the development of some diseases, and promote the proliferation of beneficial bacterial species (Oteiza et al., 2018).

However, while most studies on *A. oxyphylla* have focused on its beneficial effects on humans as traditional Chinese medicine, we aimed to explore its potential application as an additive in the animal production industry. As far as we know, little was known about the effects of AOP as feed additives or about its possible uses in the production of chickens. The purpose of this experiment was to ascertain how adding different concentrations of AOP to the diet affected the hens' immune system, microflora composition, nutrient transporters, anti-apoptotic ability, immune status, and ability to produce eggs and eggs of high quality.

## MATERIALS AND METHODS

### Preparation of AOP

*Alpiniae oxyphyllae Fructus* was provided by Anguo Chang'an Chinese Medicinal Herbs Co., Ltd (Hebei, China). Following drying, the material was ground into a powder using a pulverizer, sieved through an 80-mesh screen for filtration, and kept for later use at room temperature (25°C). Association of Official Analytical Chemists (**AOAC**) procedures were used to determine the proximate chemical composition of AOP, which includes calcium, total phosphorus, ash, crude protein, and ether extract (AOAC, 2010). The phenol-sulfuric acid method was used to identify the polysaccharides in AOP, with glucose acting as a reference (Yang et al., 2021). The NaNO_2_-Al (NO_3_)_3_-NaOH colorimetric method was used to measure the flavonoid content of AOP, with rutin acting as a reference (Park et al., 2008). Gallic acid was used as the standard in the Folin-ciocalteu method to calculate the polyphenol content of AOP (Jayaprakasha et al., 2001). The detailed nutritional levels and main bioactive compounds were presented in Table 1.

**Table 1:** The nutritive composition and main bioactive compounds in dried powder of *Alpiniae oxyphyllae Fructus*.

Nutritive ingredients	Content (%)	Bioactive compound	Content (%)
Crude protein	7.45	Polyphenols	19.85
Crude fat	2.10	Flavonoids	10.56
Crude fiber	11.10	Polysaccharides	2.06
Ash	6.70		
Ca	0.24		

### Experimental Birds, Diets, and Management

The Animal Care and Use Committee of South China Agricultural University (approval number: SYXK 2022–0136, Guangzhou, China) approved each experimental protocol.

A total of 252 healthy Wenchang breeder hens (30 wk old) were provided by Enping Jilong Industrial Co., Ltd (Jiangmen, China). The hens were evenly and randomly divided into 3 treatment groups, each consisting of 6 replicates of 14 breeder hens. The 3 groups received the following treatments: AOP1 (basal diet + 1 g/kg AOP), AOP3 (basal diet + 3 g/kg AOP), and CON (basal diet + 0 g/kg AOP). **Table S1** displayed the ingredients and composition of the diets. After a week-long acclimation period, all breeder hens were fed the designated experimental diets for a duration of 8 wk (d 56). During the trial period, each hen received 85 g of food daily at 07:30 and had unrestricted access to water. Breeder hens were artificially inseminated every 3 d with 35 μL of pooled semen per bird, as per the protocol described by (Liu et al., 2019). The chicken coop was kept at 27.5 ± 2.5 °C and 78.5 ± 3.5%, respectively, in terms of temperature and relative humidity. A lighting schedule consisting of 16 h of light and 8 h of darkness was followed throughout the experiment.

### Sample Collection

Six laying breeder hens were chosen at random from each group at the end of the trial (one per replicate). After a 12-h fast, 5 mL of blood was collected from the wing vein and centrifuged for 10 min at 4°C and 3,000 × *g*. The resultant serum was kept for additional examination at -80°C. Organ indices were determined by removing, weighing, and analyzing the liver, oviduct, and ovary tissues after euthanizing the chickens via cervical dislocation. Additionally, 2 sections of the jejunum were excised and preserved in a formalin solution for microscopic examination. Measurements were taken of the oviduct's length and the number of graded follicles, which included pre-ovulatory follicles (**POF**, >10 mm in diameter) and small yellow follicles (**SYF**, 8∼10 mm in diameter). Likewise, tissue samples from the liver and ovaries, as well as mucosal samples from the middle jejunum, were taken and preserved at -80°C for later examination. The cecal digesta samples were immediately collected on ice and stored at -80°C for 16S rRNA analysis.

### Production Performance and Egg Quality

At the beginning of 32 wk of the trial, the daily feed intake and egg production were recorded. Egg production, average egg weight, egg mass, feed intake, and feed conversion ratio (FCR, feed/egg) were calculated at the end of 40 wk.

At the conclusion of the trial, twelve randomly selected eggs from each group were analyzed for egg quality, resulting in a total of 6 replicates per group, each comprising 2 eggs. The collected eggs were assessed for Haugh unit, yolk color, albumen height, and shell strength on a level surface using an egg quality analyzer (ET-6000, Nabel, Kyoto, Japan). An electronic balance was used to calculate the weight of the egg, yolk, and eggshell. Eggshell thickness was measured with a vernier caliper (Guilin Guanglu Measuring Instrument Co., Ltd., Guangxi, P.R. China), and the long and short diameters of the eggs were measured with a spiral micrometer (217-111, Nanjing Sucian Measuring Instrument Co., Ltd., Nanjing, China).

On the last 4 d of the experiment period, 120 eggs were collected from each group and kept at a temperature of 13°C and a relative humidity of 80%. All eggs were kept in the Bengbu Sanyuan Incubation Equipment Co., Ltd. (Anhui, China) and hatched under standard conditions of 70 to 80% humidity at 37.8°C with intermittent rotation. Additionally, from the 15th d of incubation until hatching, the eggs were sprayed with water once daily (Xia et al., 2020). The number of dead embryos and fertile eggs were determined by candling eggs after 19 d of incubation. Fertility, the ability of fertile eggs to hatch, the ability of set eggs to hatch, and embryo mortality were among the measured and recorded parameters.

### Egg Yolk Fatty Acids Profile

At the conclusion of the experiment, 6 qualified eggs per group were retrieved (one egg per replicate × 6 replicates per group), and the method described by Zhang et al. (2023), was employed to assess the yolk fatty acid composition. Essentially, 200 mg of lyophilized egg yolk were mixed with 1 mL of n-hexane, 4 mL of methanolic HCl solution, and 1 mL of internal standard (1 mg/mL 11-carbon fatty acid methyl ester). Subsequently, the mixture was incubated at 80°C for 2.5 h. After cooling, the supernatant was collected following the addition of 5 mL of a 7% potassium carbonate solution. A capillary column and a flame ionization detector were fitted to a gas chromatograph (6,890 series, Agilent Technologies, Wilmington, DE) for the analysis of the fatty acids in the egg yolk.

### Serum Biochemical Parameters

A fully automated biochemistry analyzer, the Mindray BS-380 (Shenzhen Mindray Bio-Medical Electronics Co., Ltd., Shenzhen, China), along with the appropriate detection kits, were used to measure the serum concentrations of the following: albumin (**ALB**), glucose (**GLU**), calcium (**Ca**), phosphorus (**P**), triglycerides (**TG**), total cholesterol (**TC**), total protein (**TP**), albumin (**ALB**), and calcium (**Ca**).

### Determination of Serum Reproductive Hormone, Antioxidant Capacity, Immunoglobulin and Caspases Level

Using commercial kits and following the manufacturer's instructions, the activities of glutathione peroxidase (**GSH-Px**), superoxide dismutase (**SOD**), total antioxidant capacity (**T-AOC**), and the concentration of malondialdehyde (**MDA**) in the serum were measured (Jiancheng Bioengineering Institute, Nanjing, Jiangsu, China). Additionally, commercial ELISA kits from Shanghai Enzyme-linked Biotechnology Co., Ltd. (China) were utilized to quantify the content of immunoglobulin A (**IgA**), immunoglobulin M (**IgM**), caspase-8, follicle-stimulating hormone (**FSH**), and 17β-estradiol (**E2**) in the serum.

### Hematoxylin and Eosin Staining

The jejunal tissue (2 cm from the mid-jejunum) of laying breeder hens was removed and fixed in 4% paraformaldehyde. Subsequently, the tissue was embedded in paraffin, sliced, dehydrated, and stained with hematoxylin and eosin. Villus height (**VH**) and crypt depth (**CD**) were measured using microscope image processing software (Image-Pro Plus 6.0, Media Cybernetics, MD). Finally, the ratio of villus height to crypt depth (**VH/CD**) was calculated.

### Tissue RNA Extraction and qRT-PCR Analysis

Total RNA was isolated from tissues (liver, ovary, and jejunal mucosa) using Trizol (Vazyme Biotech Co., Ltd., Nanjing, China). Subsequently, cDNA synthesis was performed using the Takara PrimeScript RT Kit (Vazyme Biotech Co., Ltd., Nanjing, China). For quantitative real-time PCR (**qRT-PCR**), the ChamQ Universal SYBR qPCR Master Mix (Vazyme Biotech Co., Ltd., Nanjing, China) was utilized following the manufacturer's instructions. The primer sequences for the target genes are provided in **Table S2**. After normalization to the housekeeping gene β-actin, fold changes were calculated, and mRNA abundance was quantified using the 2^−ΔΔCt^ method (Livak and Schmittgen, 2001).

### 16S rRNA Sequencing and Gut Microbiota Analysis

A The intestinal microbiota in the cecal content samples was examined through bacterial 16S rRNA gene sequencing, as described previously (Liu et al., 2023). D As directed by the manufacturer, the QIAamp DNA Stool Kit (Qiagen, Valencia, United States) was used to extract DNA from fecal samples. The primer sequences F: ACTCCTACGGGAGGCAGCA and R: GGACTACHVGGGTWTCTAAT were used to amplify the V3–V4 region of the microbial 16S rRNA gene by PCR. Sequencing was carried out by Shanghai Paisano Biotechnology Co., Ltd. (Shanghai, China) using the Illumina MiSeq gene sequencing platform. Microbial analysis was conducted using the Genescloud Platform (www.genescloud.cn). The overall composition and relative species abundances of intestinal microbial communities were assessed using the Kruskal-Wallis test. To determine statistical significance and biological relevance, a linear discriminant analysis effect size (**LEfSe**) study was conducted. Additionally, Kyoto encyclopedia of genes and genomes (**KEGG**) metabolic pathway analysis was performed to evaluate functional differences between groups. Predictions regarding the functional differences of cecal microbiota in hens at different laying phases were made using phylogenetic investigation of communities by reconstruction of unobserved states (**PICRUSt2**) (Parks et al., 2014). The repository names and accession numbers are listed below: https://www.ncbi.nlm.nih.gov/sra/PRJNA1040004.

### Statistical Analysis

The data were examined using Duncan's multiple comparison test and one-way analysis of variance (**ANOVA**) using SPSS 22.0 statistical software (SPSS Institute Inc., Chicago, IL). Orthogonal polynomial comparisons were used to test the linear and quadratic responses to the AOP levels. Graphs were generated using GraphPad Prism 7.0 software (GraphPad; San Diego, CA). Trends were observed between *p* > 0.05 and *p* < 0.10, with significant findings defined as *p* < 0.01 or *p* < 0.05.

## RESULTS

### Production Performance of Hens

Table 2 displays the impact of dietary AOP on laying breeder hens' production performance. The feed conversion ratio of the hens dropped linearly (*p* < 0.05) as the inclusion of AOP increased in comparison to CON group. Moreover, with increasing levels of supplemented AOP, the laying rate and average egg weight of laying breeder hens increased linearly (*p* < 0.01). However, supplemental AOP had no significant effect on the proportion of qualifying eggs in hens (*p* > 0.05).

**Table 2:** Effects of dietary AOP supplementation on the production performance of laying breeder hens.

Items	Treatments			SEM	*p*-value		
	CON	AOP1	AOP3		AOP	Linear	Quadratic
Laying rate (%)	63.52^b^	64.28^b^	66.36^a^	0.448	0.016	0.006	0.403
Average egg weight (g)	43.77^b^	44.22^ab^	44.82^a^	0.017	0.023	0.007	0.802
Percentage of qualified eggs (%)	95.51	95.92	96.30	0.252	0.465	0.224	0.983
Feed conversion ratio (g/g)	3.51^a^	3.29^b^	3.24^b^	0.047	0.039	0.018	0.320

a-b Means within a row with no common superscript differ significantly (*p* < 0.05). Values are mean ± SEM of 84 hens. Abbreviations: CON, control group, basal diet; AOP1, basal diet supplemented with 1 g/kg AOP; AOP3, basal diet supplemented with 3 g/kg AOP.

### Fertilizing Capacity and Hatchability of Hens

Table 3 shows the breeder hens' capacity for fertilization and hatchability. Supplementation with higher doses of AOP resulted in a significant linear decrease in embryo mortality (*p* < 0.01) and a linear increase in viable egg hatching and set egg hatchability (*p* < 0.05). However, there were no significant variations in the fertility of laid eggs in hens across the treatment groups (*p* > 0.05).

**Table 3:** Fertilizing capacity and hatchability of laying breeder hens.

Items	Treatments			SEM	*p*-value		
	CON	AOP1	AOP3		AOP	Linear	Quadratic
Embryo mortality (%)	6.30^a^	4.39^ab^	1.75^b^	0.687	0.014	0.004	0.759
Fertility of set eggs (%)	92.50	94.17	95.00	0.953	0.580	0.313	0.844
Hatch of fertile eggs (%)	93.70^b^	95.61^ab^	98.25^a^	0.687	0.014	0.004	0.759
Hatchability of set eggs (%)	86.67^b^	90.00^ab^	93.33^a^	1.143	0.048	0.015	1.000

a-c Means within a row with no common superscript differ significantly (*p* < 0.05). Values are means ± SEM (n = 4 plates of 30 eggs each). Abbreviations: CON, control group, basal diet; AOP1, basal diet supplemented with 1 g/kg AOP; AOP3, basal diet supplemented with 3 g/kg AOP.

### Reproductive Organs and Ovarian Follicle Development of Hens

Table 4 illustrates that the oviduct weight, number of SYF, and number of POF increased linearly (*p* < 0.05), while the oviduct index responded quadratically (*p* < 0.001). However, there were no significant variations observed in the ovarian weight, ovary index, oviduct length, or liver index between the 3 groups (*p* > 0.05).

**Table 4:** Effects of dietary AOP on the reproductive organ and ovarian follicle development in laying breeder hens.

Items	Treatments			SEM	*p*-value		
	CON	AOP1	AOP3		AOP	Linear	Quadratic
Liver index (%)	1.81	1.88	1.82	0.053	0.852	0.943	0.581
Oviduct weight (g)	37.47^b^	44.90^a^	45.45^a^	1.222	0.004	0.003	0.092
Oviduct index (%)	1.89^c^	2.67^a^	2.28^b^	0.097	0.001	0.023	0.001
Oviduct length (cm)	51.00	54.67	54.83	1.406	0.480	0.289	0.571
Ovarian weight (g)	41.13	41.30	43.55	1.607	0.809	0.566	0.774
Ovary index (%)	2.07	2.20	2.18	0.082	0.815	0.605	0.718
Number of SYF	12.83^b^	17.33^a^	17.83^a^	0.878	0.026	0.014	0.218
Number of POF	5.00^b^	6.83^ab^	7.50^a^	0.422	0.031	0.012	0.453

a-c Means within a row with no common superscript differ significantly (*p* < 0.05). Values are means ± SEM (n = 6 hens). Abbreviations: CON, control group, basal diet; AOP1, basal diet supplemented with 1 g/kg AOP; AOP3, basal diet supplemented with 3 g/kg AOP; POF, preovulatory follicles; SYF, small yellow follicles.

### Egg Quality of Laying Hens

Table 5 lists the egg quality of hens fed AOP. The egg shape index, yolk color, yolk percentage, eggshell thickness, eggshell ratio, and eggshell ratio did not significantly change with the introduction of AOP (*p* > 0.05). In addition, AOP inclusion resulted in significant linear and quadratic increases (*p* < 0.05) in eggshell strength, albumen height, and Haugh unit.

**Table 5:** Egg quality of laying breeder hens.

Items	Treatments			SEM	*p*-value		
	CON	AOP1	AOP3		AOP	Linear	Quadratic
Egg shape index	1.30	1.31	1.27	0.008	0.217	0.169	0.276
Eggshell thickness, mm	0.312	0.308	0.314	0.004	0.392	0.813	0.618
Eggshell strength (kg/cm^2^)	3.50^b^	4.58^a^	4.28^a^	0.159	0.013	0.006	0.021
Eggshell ratio (%)	12.70	13.33	13.46	0.192	0.232	0.113	0.529
Albumen height (mm)	2.57^b^	3.78^a^	3.35^a^	0.173	0.011	0.048	0.019
Yolk colour	5.33	5.83	5.67	0.184	0.541	0.469	0.404
Haugh unit	48.74^b^	62.87^a^	57.11^a^	1.870	0.005	0.045	0.007
Yolk percentage (%)	32.65	33.02	32.78	0.444	0.943	0.903	0.751

a-b Means within a row with no common superscript differ significantly (*p* < 0.05). Values are means ± SEM (n = 12 eggs). Abbreviations: CON, control group, basal diet; AOP1, basal diet supplemented with 1 g/kg AOP; AOP3, basal diet supplemented with 3 g/kg AOP.

### Fatty Acid Content of Egg Yolk

Table 6 shows the fatty acid content of egg yolks from laying breeder hens fed AOP. As AOP inclusion increased relative to the CON group, the levels of palmitic acid (**PA**, C16:0) and total saturated fatty acids (**SFA**) decreased (*p* < 0.001) both linearly and quadratically. Additionally, there was a linear decrease in behenic acid (**BA**, C22:0), a type of SFA, in the AOP group (*p* < 0.01). The levels of monounsaturated fatty acids (**MUFA**) and polyunsaturated fatty acids (**PUFA**) in the egg yolk were similar across the 3 groups (*p* > 0.05).

**Table 6:** Fatty acid content in egg yolk of laying breeder hens.

Items	Treatments			SEM	*p*-value		
	CON	AOP1	AOP3		AOP	Linear	Quadratic
Saturated fatty acid, %							
C14:0	0.047	0.036	0.048	0.004	0.369	0.824	0.114
C15:0	0.007	0.004	0.006	0.001	0.330	0.454	0.205
C16:0	2.316^a^	0.744^b^	0.342^b^	0.252	<0.001	<0.001	0.037
C17:0	0.025	0.020	0.027	0.002	0.430	0.829	0.218
C18:0	1.624	1.245	1.638	0.111	0.282	0.959	0.119
C22:0	0.017^a^	0.013^ab^	0.007^b^	0.002	0.017	0.006	0.789
Total SFA	4.037^a^	2.062^b^	2.067^b^	0.301	0.001	0.001	0.025
Monounsaturated fatty acid, %							
C14:1	0.008	0.007	0.006	0.001	0.645	0.399	0.709
C16:1	0.375	0.347	0.321	0.025	0.699	0.407	0.986
C17:1	0.010	0.008	0.010	0.001	0.457	0.897	0.223
C18:1n-9	6.760	6.386	6.726	0.473	0.947	0.979	0.747
C20:1	0.072	0.060	0.064	0.006	0.723	0.597	0.554
C24:1	0.039	0.044	0.052	0.005	0.619	0.343	0.886
Total MUFA	7.263	6.851	7.718	0.500	0.947	0.950	0.751
Polyunsaturated fatty acid, %							
C20:2	0.019	0.020	0.018	0.002	0.834	0.769	0.607
C18:3n-3	0.025	0.023	0.022	0.002	0.834	0.565	0.894
C20:3n-3	0.262	0.117	0.189	0.033	0.214	0.359	0.132
C20:5n-3	0.002	0.003	0.004	0.001	0.727	0.438	0.918
C22:6n-3	0.222	0.196	0.212	0.020	0.881	0.849	0.649
C18:2n-6	2.498	2.330	2.458	0.202	0.947	0.941	0.752
C18:3n-6	0.022	0.018	0.021	0.002	0.699	0.869	0.416
C20:3n-6	0.010	0.013	0.011	0.002	0.768	0.843	0.494
C20:4n-6	0.101	0.211	0.141	0.029	0.321	0.581	0.166
Total PUFA	3.161	2.931	3.075	0.249	0.939	0.898	0.748
n-3	0.512	0.339	0.426	0.048	0.374	0.484	0.228
n-6	2.630	2.572	2.631	0.219	0.993	1.000	0.908
n-6/n-3	5.784	7.571	7.172	0.641	0.524	0.404	0.447

a-b Means within a row with no common superscript differ significantly (*p* < 0.05). Values are means ± SEM (n = 6 eggs). Abbreviations: CON, control group, basal diet; AOP1, basal diet supplemented with 1 g/kg AOP; AOP3, basal diet supplemented with 3 g/kg AOP; SFA, saturated fatty acid; MUFA, monounsaturated fatty acid; PUFA, polyunsaturated fatty acid.

### Serum Biochemical Index of Hens

Table 7 shows that AOP had no significant effect on serum AST and ALT concentrations (*p* > 0.05). However, serum concentrations of ALB and P increased linearly (*p* < 0.05) with increasing AOP inclusion, while serum concentrations of TC, TG, and GLU decreased linearly (*p* < 0.05). Serum concentrations of Ca tended to increase linearly with increasing AOP inclusion (*p* = 0.062). Furthermore, there was a noteworthy rise in serum TP contents (*p* < 0.05) in the AOP1 group when compared with the CON group.

**Table 7:** Effects of dietary AOP supplementation on the serum biochemical index in laying breeder hens.

Items	Treatments			SEM	*p*-value		
	CON	AOP1	AOP3		AOP	Linear	Quadratic
AST (U/L)	236.07	222.70	211.88	7.059	0.398	0.183	0.933
ALT (U/L)	4.92	4.08	3.75	0.470	0.608	0.341	0.811
ALB (g/L)	10.78^b^	13.62^a^	13.42^a^	0.539	0.045	0.036	0.146
TP (g/L)	43.58^b^	53.07^a^	47.95^ab^	1.415	0.013	0.134	0.008
TC (mmol/L)	4.65^a^	2.74^b^	2.87^b^	0.346	0.025	0.018	0.132
TG (mmol/L)	11.38^a^	4.61^b^	5.49^b^	0.882	<0.001	<0.001	0.004
GLU (mmol/L)	6.08^a^	4.64^b^	4.20^b^	0.309	0.022	0.009	0.370
Ca (mmol/L)	3.63	3.98	3.92	0.067	0.059	0.062	0.115
P (mmol/L)	2.83^b^	3.75^a^	3.76^a^	0.166	0.020	0.014	0.135

a-b Means within a row with no common superscript differ significantly (*p* < 0.05). Values are means ± SEM (n = 6 hens). Abbreviations: CON, control group, basal diet; AOP1, basal diet supplemented with 1 g/kg AOP; AOP3, basal diet supplemented with 3 g/kg AOP; AST, aspartate amino transferase; ALT, alanine transaminase; ALB, albumin; TP, total protein; TC, total cholesterol; TG, triglyceride; GLU, glucose; Ca, calcium; P, phosphorus.

### Reproductive Hormone Indices of Hens

**Figure 1:**
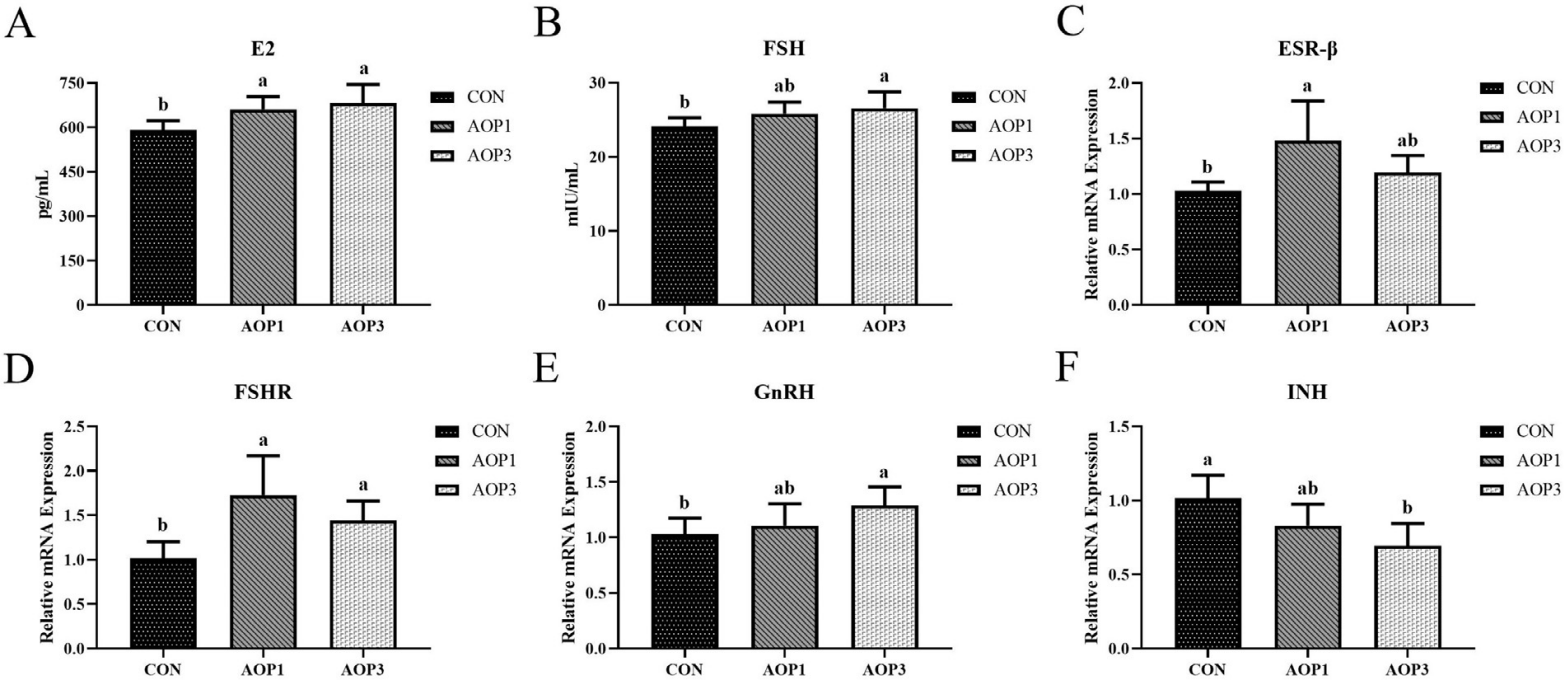
Effects of dietary supplementation with AOP on hormone indices of laying breeder hens. (A) Serum E2. (B) serum FSH. (C–F) The mRNA expression of hormone-related genes (ESR-β, FSHR, GnRH, and INH) in ovary. Different lowercase letters in the figure indicate statistically significant differences (*p* < 0.05, n = 6). Abbreviations: CON, control group, basal diet; AOP1, basal diet supplemented with 1 g/kg AOP; AOP3, basal diet supplemented with 3 g/kg AOP; E2, 17β-estradiol; FSH, follicle-stimulating hormone; ESR-β, estrogen receptor beta; FSHR, follicle-stimulating hormone receptor; GnRH, gonadotropin-releasing hormone; INH, inhibit 1.

The effects of dietary supplements with AOP on serum hormone levels and the mRNA expression of hormone-related genes were shown in Figure 1. Regarding serum hormone levels, the contents of serum E2 and FSH increased linearly in laying breeder hens as the dietary levels of AOP increased (*p* < 0.05). Next, we examined the mRNA expression of hormone-related genes in the ovary. There was a significant linear and quadratic increase (*p* < 0.05) in the relative mRNA expression of FSHR and a linear increase (*p*  < 0.05) in the expression of GnRH in the ovary with increasing levels of AOP in the diets. Furthermore, there was a linear down-regulation (*p* < 0.01) of the relative mRNA expression of INH in the ovary with AOP food supplementation. Additionally, the mRNA expression of ESR-β in the AOP group at 1 g/kg was significantly higher than that in the CON group (*p* < 0.05).

### Antioxidant Capacity of Hens

**Figure 2:**
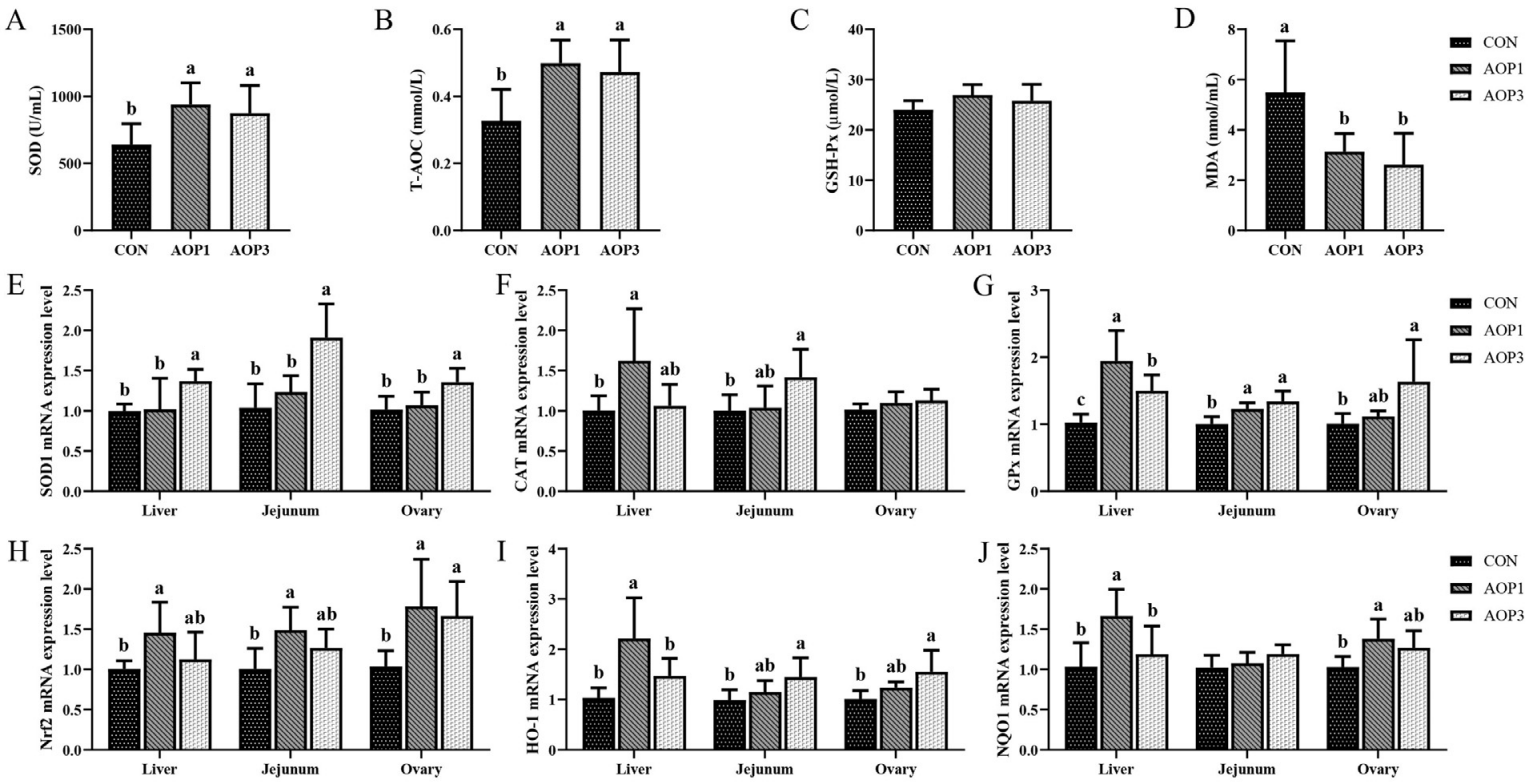
Effects of dietary supplementation with AOP on the antioxidant capacity of laying breeder hens. (A–D) Serum SOD, T-AOC, GSH-Px, and MDA. (E–J) The mRNA expression of antioxidant-related genes (SOD1, GPx, CAT, Nrf2, HO-1, and NQO1) in the liver, jejunum, and ovary, respectively. Different lowercase letters in the figure indicate statistically significant differences (*p* < 0.05, n = 6). Abbreviations: CON, control group, basal diet; AOP1, basal diet supplemented with 1 g/kg AOP; AOP3, basal diet supplemented with 3 g/kg AOP; SOD, superoxide dismutase; T-AOC, total antioxidant capacity; GSH-Px, glutathione peroxidase; MDA, malondialdehyde; SOD1, copper and zinc superoxide dismutase; GPx, glutathione peroxidase; CAT, catalase; Nrf2, nuclear factor erythroid2-related factor 2; HO-1, heme oxygenase 1; NQO1, NAD(P)H quinone oxidoreductase 1.

The effects of dietary supplementation with AOP on the antioxidant function of laying breeder hens were presented in Figure 2. The activity of serum SOD and T-AOC showed a linear increase (*p* < 0.05) with increasing levels of added AOP, while the MDA content exhibited a linear decrease (*p*  < 0.05). However, there were no significant changes (*p* > 0.05) observed in the activity of serum GSH-Px. Additionally, we investigated the mRNA expression of genes associated with antioxidants in laying breeder chickens. The relative mRNA expression of SOD1 and GPx in the liver was linearly up-regulated (*p* < 0.05) by dietary supplementation with AOP, with this effect observed across increasing levels of AOP addition. Moreover, there was a significant linear increase (*p*  < 0.05) in the relative mRNA expression of GPx, HO-1, SOD1, and CAT in the jejunum. When AOP was added to the diet at a rate of 1 g/kg, the relative mRNA expression of CAT, Nrf2, HO-1, and NQO1 in the liver, as well as Nrf2 expression in the jejunal mucosa, increased significantly (*p* < 0.05) compared to the CON group. Moreover, the relative mRNA expression of SOD1, GPx, Nrf2, and HO-1 exhibited a linear increase (*p* < 0.05) with increasing levels of AOP inclusion. Furthermore, there was a trend for NQO1 expression to increase linearly (*p*= 0.083) in the ovary. However, when AOP was added, there were no discernible effects (*p*> 0.05) on the relative mRNA expression of NQO1 in the mucosa of the jejunum and CAT in the ovaries of laying breeder hens.

### Anti-Apoptosis Ability of Hens

**Figure 3:**
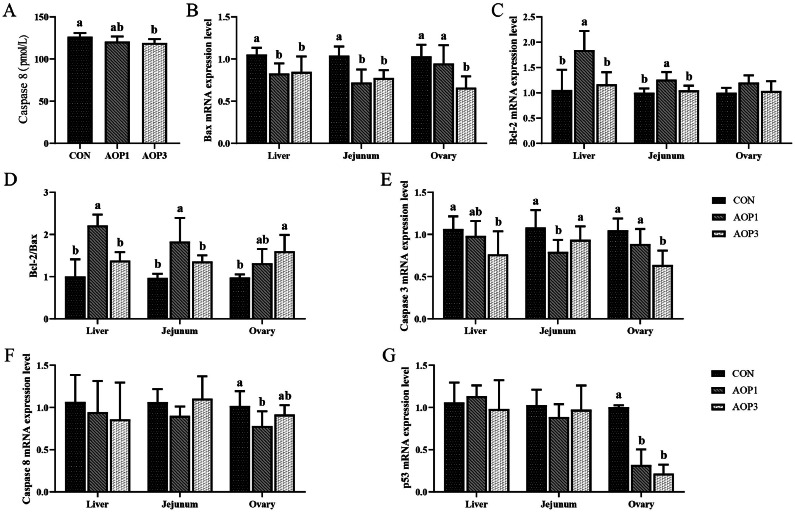
Effects of dietary supplementation with AOP on anti-apoptotic ability in laying breeder hens. (A) Serum caspase-8. (B–G) The mRNA expression of apoptosis-related genes (Bax, Bcl-2, Bcl-2/Bax, Caspase 3, Caspase 8, and p53) in liver, jejunum, and ovary, respectively. Different lowercase letters in the figure indicate statistically significant differences (*p* < 0.05, n = 6). Abbreviations: CON, control group, basal diet; AOP1, basal diet supplemented with 1 g/kg AOP; AOP3, basal diet supplemented with 3 g/kg AOP; Bax, bcl2-associated x; Bcl-2, b-cell lymphoma-2.

The effect of dietary supplementation with AOP on apoptosis indices within the laying breeder hens was shown in Figure 3. When increasing the amount of AOP supplementation, there was a significant linear decrease (*p *< 0.05) in the blood levels of caspase-8 in laying breeder hens. Additionally, we examined the mRNA expression of genes linked to apoptosis in laying breeder hens. AOP levels linearly altered the ratio of Bcl-2/Bax (*p* = 0.067), and the mRNA expressions of Bax and Caspase3 in the liver exhibited a linear decrease with increasing AOP levels (*p* < 0.05). Additionally, there was a linear decrease in the Bcl-2/Bax ratio (*p* = 0.093) and a linear decrease in the expression of Bax in the jejunal mucosa (*p* < 0.05) with increasing AOP levels. Furthermore, increasing AOP inclusion resulted in a linear decrease (*p* < 0.05) in mRNA expressions of Bax, Caspase3, and p53 in the ovary, while the Bcl-2/Bax ratio increased linearly (*p* < 0.05). Compared to the CON group, dietary AOP at 1 g/kg increased (*p* < 0.05) Bcl-2 expression in the liver and jejunal mucosa but reduced Caspase3 expression in the jejunal mucosa, as well as Caspase8 expression in the ovary. However, AOP supplementation had no discernible impact on the expression of Bcl-2 in the ovaries, as well as the expression of caspase8 and p53 in the liver and jejunal mucosa (*p* > 0.05).

### Immune Function of Hens

**Figure 4:**
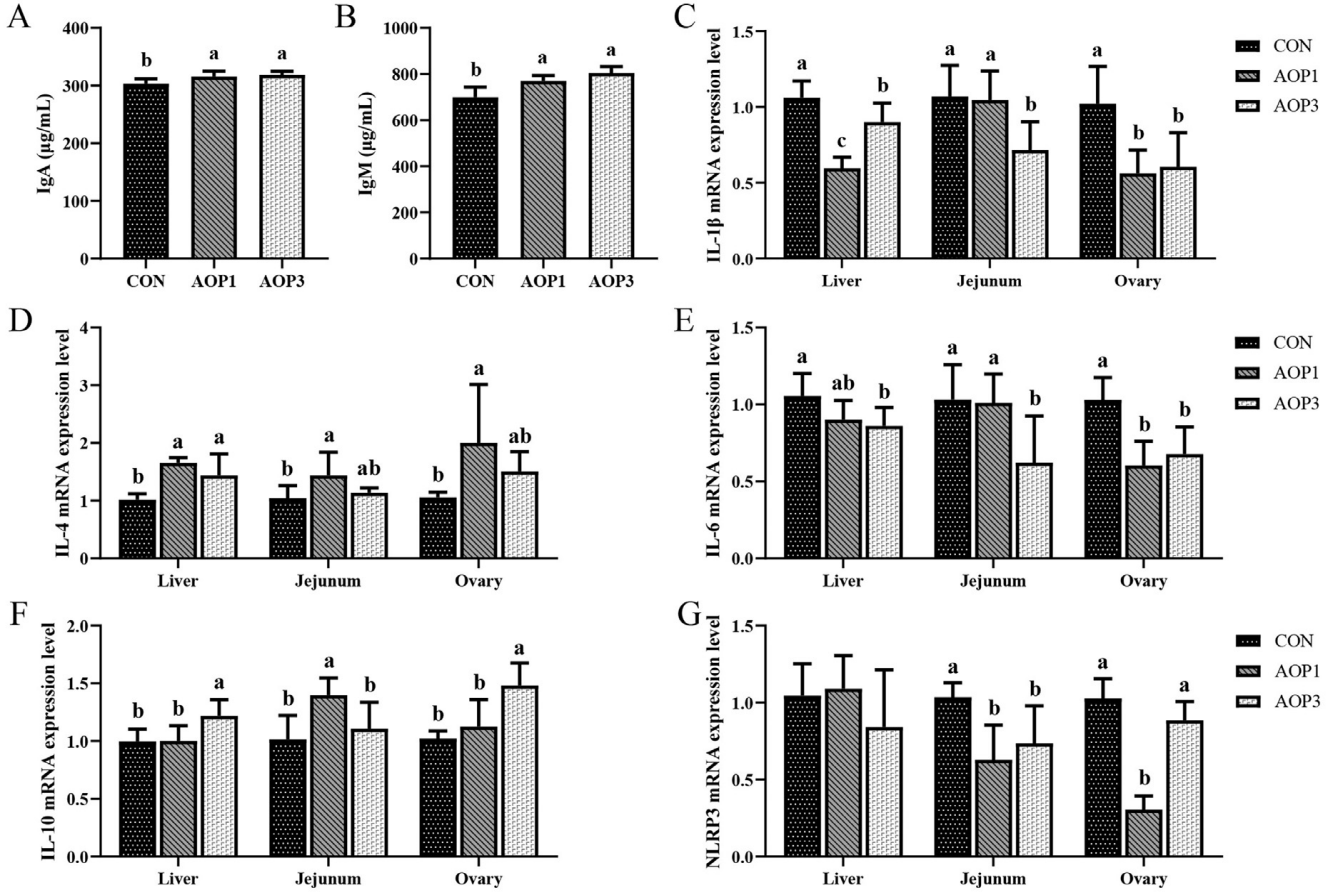
Effects of dietary supplementation with AOP on the immune function in laying breeder hens. (A–B) Serum immunoglobulin A (IgA) and immunoglobulin M (IgM). (C–G) The mRNA expression of inflammatory related genes (IL-1β, IL-4, IL-6, IL-10, and NLRP3) in liver, jejunum, and ovary, respectively. Different lowercase letters in the figure indicate statistically significant differences (*p* < 0.05, n = 6). Abbreviations: CON, control group, basal diet; AOP1, basal diet supplemented with 1 g/kg AOP; AOP3, basal diet supplemented with 3 g/kg AOP; IgA, immunoglobulin A; IgM, immunoglobulin M; IL-1β, interleukin-1β; IL-4, interleukin-4; IL-6, interleukin-6; IL-10, interleukin-10; NLRP3, NOD-like receptor protein 3.

The effects of dietary supplementation with AOP on immune function in laying breeder hens were detailed in Figure 4. The content of serum IgA and IgM was enhanced by dietary AOP in a linear manner (*p* < 0.05) in proportion to serum immunoglobulin levels. Additionally, the mRNA expressions of IL-4 and IL-10 in the liver were considerably elevated (linear, *p* < 0.05) by dietary supplementation with AOP, whereas the mRNA expressions of IL-1β and IL-6 were down-regulated (linear, *p* < 0.05) with increasing AOP levels. However, NLRP3 expression in the liver did not significantly alter (*p* > 0.05). Additionally, as dietary AOP supplementation increased, the mRNA expressions of NLRP3, IL-6, and IL-1β in the jejunal mucosa decreased linearly (*p* < 0.05). Moreover, with increasing AOP levels, the mRNA expression of IL-β and IL-6 in the ovary was linearly down-regulated (*p* < 0.05), while the mRNA expression of IL-10 was linearly up-regulated (*p* < 0.05) by AOP supplementation. Specifically, dietary supplementation with AOP at 1 g/kg increased the expression of IL-10 in the jejunal mucosa and IL-4 in the liver and jejunal mucosa compared to the CON group, while it decreased the expression of NLRP3 in the ovary (*p* < 0.05).

### Intestinal Morphology and Barrier Genes of Hens

**Figure 5:**
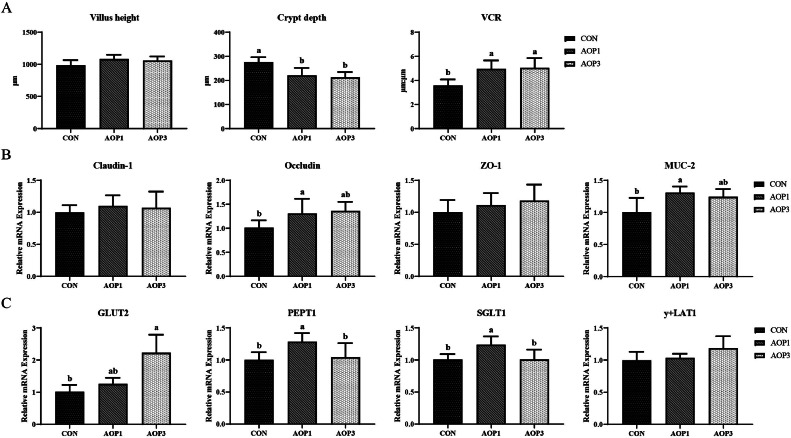
Effects of dietary supplementation with AOP on intestinal morphology and barrier genes and nutrient transport in laying breeder hens. (A) Villus height, crypt depth, and villus height to crypt depth ratio of the jejunum. (B) The mRNA expression of tight junction proteins and mucin (Claudin-1, Occludin, ZO-1, and MUC-2) in the jejunum. (C) The mRNA expression of nutrient transport related genes (GLUT2, PEPT1, SGLT1, and y+LAT1) in the jejunum. Different lowercase letters in the figure indicate statistically significant differences (*p* < 0.05, n = 6). Abbreviations: CON, control group, basal diet; AOP1, basal diet supplemented with 1 g/kg AOP; AOP3, basal diet supplemented with 3 g/kg AOP; ZO-1, zonula occludens-1; MUC-2, mucin-2; GLUT2, glucose transporter 2; PEPT1, peptide-transporter 1; SGLT1, sodium glucose cotransporter 1; y+LAT1, y(+)L-type amino acid transporter 1.

The effect of dietary supplementation with AOP on the jejunal morphology of laying breeder hens was presented in **Figure S1**. Additionally, in order to examine the effect of AOP on barrier function, we assessed the mRNA expression in the jejunal mucosa of tight junction proteins, mucin, and genes related to nutrient transport (Figure 5). While there were no significant changes (*p* > 0.05) in the height of the jejunal villi in laying breeder hens, there was a linear increase (*p* < 0.05) in the villus/crypt ratio (**VCR**) and a decrease (*p* < 0.05) in the depth of the jejunal crypt (Figure 5A). Additionally, there was a linear increase (*p* < 0.05) in the expression of jejunal occludin (Figure 5B) and in the expression of jejunal GLUT-2 and y+LAT1 (Figure 5C). The mRNA expression of mucin-2 (**MUC-2**), peptide transporter 1 (**PEPT1**), and sodium/glucose cotransporter 1 (**SGLT1**) in the jejunal mucosa was upregulated (*p* < 0.05) with dietary AOP inclusion at a level of 1 g/kg compared to the CON group. However, there were no significant differences in the mRNA expression of Claudin-1, ZO-1, or y+LAT1 between the treatment groups (*p* > 0.05).

### Microbial Diversity of Cecal Digesta

**Figure 6:**
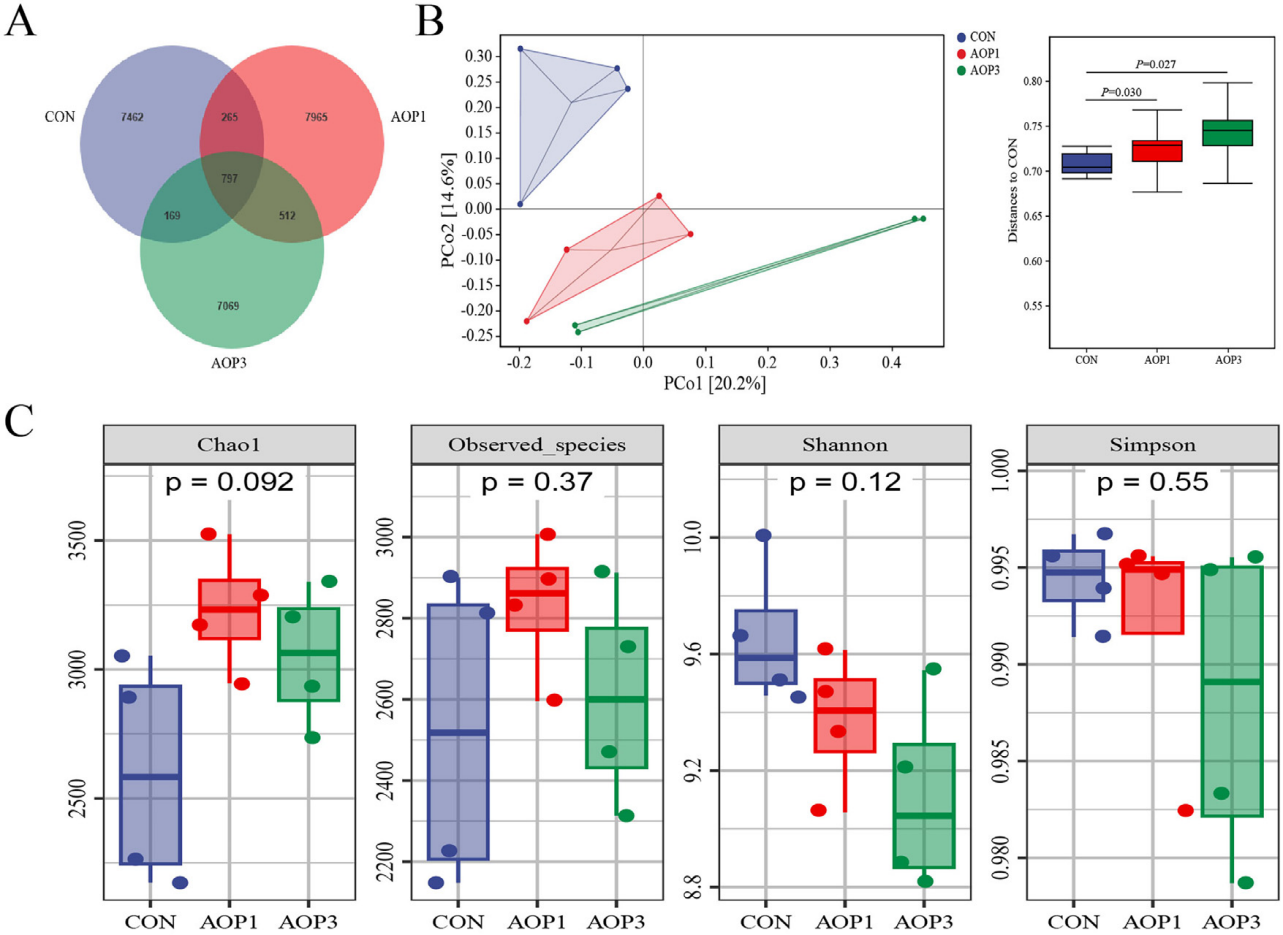
Effects of AOP on cecal microbiota diversity in laying breeder hens. (A)Veen diagram. (B) Principal component analysis (**PCA**) scatterplot. (C) Bacterial alpha-diversity indices (Chao1, Observed_species, Shannon, and Simpson). * indicates a significant difference at *p* < 0.05 (n = 4). Abbreviations: CON, control group, basal diet; AOP1, basal diet supplemented with 1 g/kg AOP; AOP3, basal diet supplemented with 3 g/kg AOP.

The sequencing analysis of fecal samples from each of the 3 groups revealed an average of 90,288 raw data per sample. After quality trimming and chimera checking, 52,747 high-quality sequences were obtained from each sample. Microbial diversity in the cecal digesta is affected by AOP, as shown in Figure 6. The Venn diagram analysis revealed 8,693 operational taxonomic units (**OTU**) in the CON group, while there were 9,539 and 8,547 OTUs in the AOP1 and AOP3 groups, respectively (Figure 6A). Out of these OTUs, 797 were shared by all 3 groups, indicating a degree of similarity in the microbial composition across the samples. The principal component analysis (**PCA**) plot demonstrated a significant distinction between the CON and AOP groups (CON vs. AOP1, *R^2^* = 0.635, *p* = 0.030; CON vs. AOP3, *R^2^* = 0.688, *p* = 0.027), indicating a notable influence of AOP supplementation on the intestinal microbiota (Figure 6B). When comparing the AOP1 and AOP3 groups to the CON group, there was a trend toward an increase in the Chao1 index of cecal bacterial microbiota, as indicated by the alpha diversity index (*p* = 0.092, Figure 6C). However, there were no significant differences (*p* >0.05) observed between the 3 groups in terms of the other index, according to the study's findings.

### Composition of Cecal Digesta

**Figure 7:**
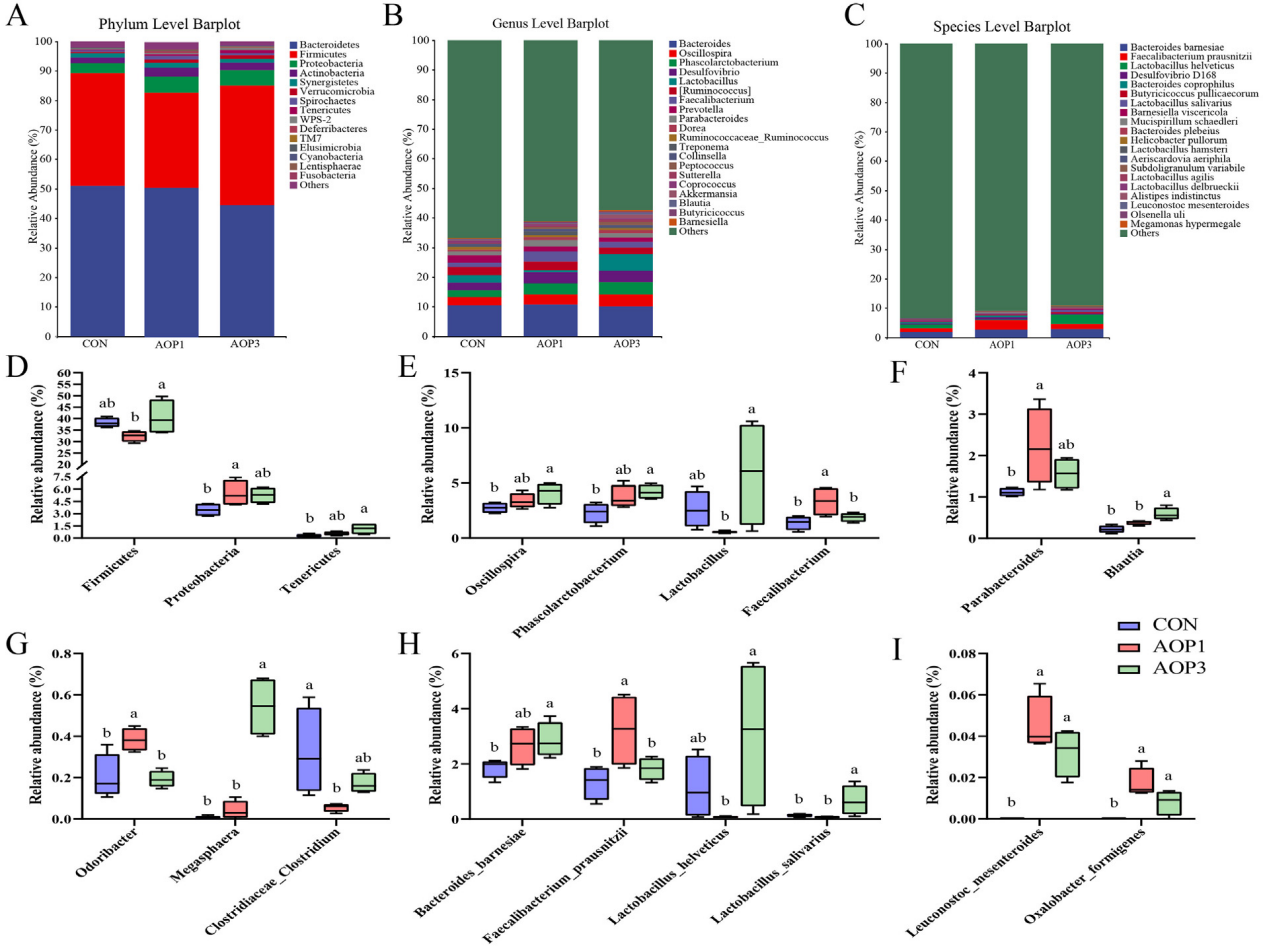
Effects of dietary supplementation with AOP on the composition of the cecal microbiota in laying breeder hens. (A) Phylum level barplot. (B) Genus level barplot. (C) Species level barplot. Comparison of dominant cecal microbiota at phylum (D), genus (E–G), and species (H-I) levels. Different lowercase letters in the figure indicate statistically significant differences (*p* < 0.05, n = 4). Abbreviations: CON, control group, basal diet; AOP1, basal diet supplemented with 1 g/kg AOP; AOP3, basal diet supplemented with 3 g/kg AOP.

**Figure 8:**
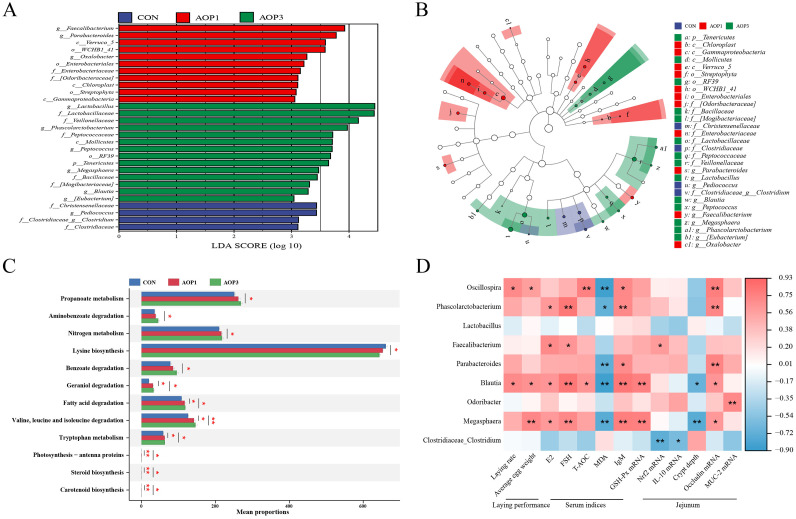
Changes in laying breeder hens cecal microbiota and functional prediction with AOP supplementation. (A) LEfSe bar based on phylum to genus level (LDA > 3). (B) Branching diagram of evolution scatterplot. (C) Functional profiles of the gut microbiota. (D) Spearman's correlation analysis between cecal microbiota and different indicators (laying performance, serum indices, and jejunal mucosa). * indicates a significant difference at *p* < 0.05 and ** indicates *p* < 0.01 (n = 4). Abbreviations: CON, control group, basal diet; AOP1, basal diet supplemented with 1 g/kg AOP; AOP3, basal diet supplemented with 3 g/kg AOP.

Figure 7 showed the composition of the microbiota at the phylum, genus, and species levels. *Bacteroidetes, Firmicutes*, and *Proteobacteria* were consistently found to be the 3 most prevalent bacterial phyla in each group of laying breeder hens (Figure 7A). *Proteobacteria* in the AOP1 group and *Tenericutes* in the AOP3 group had significantly higher relative abundances at the phylum level when compared to the CON group, as shown in Figure 7D (*p* < 0.05). Upon further examination at the genus level, we observed 20 bacteria with varying relative abundances (Figure 7B). AOP1 supplementation dramatically reduced the abundance of *Clostridiaceae_Clostridium* (*p* < 0.05) in the diet compared to the CON group but increased the abundance of *Odoribacter, Paeabacteroides*, and *Faecalibacterium* (*p* < 0.05). Similarly, the AOP3 group exhibited a significant increase in the abundance of *Blautia, Megasphaera, Oscillospira*, and *Phascolarctobacterium* (Figures 7E–7G)*.* At the species level (Figure 7C), the AOP1 group showed an overrepresentation of *Faecalibaterium prausnitzii* (*p* < 0.05), while the AOP3 group exhibited an overrepresentation of *Bacteroides barnesiae* and *Lactobacillus salvarius* (*p* < 0.05). Furthermore, both the AOP1 and AOP3 groups demonstrated significantly higher relative abundances of *Leuconostoc_mesenteroides* and *Oxalobacter_ formigenes* in comparison to CON group (*p* < 0.05) (Figures 7H–7I). We employed linear discriminant analysis effect size (**LEfSe**) analysis (LDA score > 3) to pinpoint bacteria exhibiting notable variations between the 3 groups, aiming to explore and identify distinct bacterial taxa (Figure 8A). *Christensenellaceae* (family), *Pediococcus* (genus), and *Clostridium* (family) were more abundant in the CON group; *Faecalibacterium* (genus), *Parabacteroides* (genus), and *Verruco_5* (class) were higher abundant in the AOP1 group; *Lactobacillus* (genus), *Lactobacillaceae* (family), and *Veillonellaceae* (family) were enriched in the AOP3 group. The cladogram subsequently depicted abundance values based on LEfSe, showcasing the taxa. Potential biomarkers were identified at 4, 11, and 14 distinct taxonomic levels of microorganisms in the CON, AOP1, and AOP3 groups, respectively (Figure 8B).

### Prediction of Cecal Microbial Function

Fourteen metabolic pathways (at level 3) were influenced among the groups, as depicted in Figure 8C. These pathways encompassed 3 pathways for amino acid metabolism, 2 for energy metabolism, 2 for lipid metabolism, 2 for xenobiotic biodegradation and metabolism, 2 for terpenoid and polyketide metabolism, and one for carbohydrate metabolism. Furthermore, with the application of dietary AOP3, microbial gene abundance associated with energy metabolism (nitrogen metabolism), xenobiotic biodegradation and metabolism (aminobenzoate degradation and benzoate degradation), and carbohydrate metabolism (propanoate metabolism) significantly increased (*p* < 0.05) compared to the CON group. Conversely, it decreased (*p* < 0.05) in relation to amino acid metabolism (lysine biosynthesis). The metabolism of terpenoids and polyketides (including geraniol degradation and carotenoid biosynthesis), energy metabolism (including photosynthesis-antenna proteins), lipid metabolism (including fatty acid degradation and steroid biosynthesis), and amino acid metabolism (including tryptophan metabolism and valine, leucine, and isoleucine degradation) pathways were all enhanced (*p* < 0.05) when AOP1 and AOP3 were added to the diet of laying breeder hens.

### Correlation Analysis of Altered Cecal Bacteria With Measured Parameters

The potential relationship of modifications in cecal microbiota at the genus level with laying performance, hormone indices, immune-related factors, antioxidant factors, and barrier function‐related factors was examined by applying Spearman correlation analysis (Figure 8D). Correlation analysis revealed significant associations between laying performance and the genera *Oscillospira, Blautia*, and *Megasphaera* (*p* < 0.05). Furthermore, the concentration of serum hormones (E2 and FSH) was strongly positively correlated with the genera *Phascolarctobacterium, Fecalibacterium, Blautia*, and *Megasphaera* (*p* < 0.05). Additionally, antioxidant and immune-related factors exhibited potential correlations with *Oscillospira, Phascolarctobacterium, Parabacteroides, Blautia*, and *Megasphaera*, while demonstrating a negative relationship with *Clostridiaceae_Clostridium* (*p* < 0.05). The barrier function‐related factors were possibly correlated with *Blautia* and *Megasphaera*, in addition to being positively correlated with *Oscillospira, Phascolarctobacterium, Parabacteroides*, and *Odoribacter* (*p* < 0.05).

## DISCUSSION

*Alpinia oxyphylla* was an edible and medicinal plant with a diverse range of pharmacological effects and active ingredients. These effects include antioxidant, anti-inflammatory, anti-parasitic, and neuroprotective qualities (Zhang et al., 2018). Our recent study revealed that laying breeder hens fed AOP diets exhibited a reduced feed conversion ratio. This effect could be attributed to the capacity of *A. oxyphylla* to enhance digestion, thereby improving the gastrointestinal tract's ability to absorb and utilize nutrients. Moreover, significant enhancements were observed in the average egg weight, hatchability of set eggs, laying rate, and hatchability of viable eggs. However, the inclusion of 3 g/kg AOP in the diet led to a decrease in embryo mortality. According to (Yuan et al., 2016), incorporating tea polyphenols into the diet of layer chickens enhances laying hen productivity, egg quality, and resilience to adverse effects. In our study, *Alpiniae oxyphyllae fructus* powder contained a significant proportion of polyphenols (19.85%), followed by flavonoids (10.56%) and polysaccharides (2.06%). Hence, we hypothesized that the synergistic interaction among these compounds contributed to the beneficial effects of AOP on laying hen performance.

In laying hen production systems, egg quality was paramount, as it directly impacts customer acceptance and financial profitability (Korver, 2023). The notable increase in albumen height and Haugh unit observed in the AOP group could be attributed to the presence of polyphenolic components, showcasing the beneficial impact of dietary AOP inclusion on protein anabolism (Yuan et al., 2016). Similarly, significant reductions were observed in serum TC, TG, and GLU levels following AOP treatment (Ghasemi and Nari, 2019). Nootkatone, mainly from *Alpiniae Oxyphyllae Fructus*, was shown to dramatically increase glucose consumption and decrease intracellular TG accumulation in L02 cells; this was supported by in vitro data in a study conducted by (Yong et al., 2022). The incorporation of AOP into the diet at a concentration of 3 g/kg for laying hens was observed to have advantageous impacts on both the internal and external quality of eggs. Specifically, while serum Ca concentrations exhibited a tendency to increase linearly, there were elevations in eggshell strength and serum P concentrations in the AOP-supplemented diet. Phosphorus was a crucial nutrient required by chickens, participating in numerous metabolic processes, as highlighted in this study (Bello et al., 2020). Both calcium and phosphorus were indispensable for skeletal function and eggshell formation and maintenance (Bello et al., 2020; Zhang et al., 2023). The addition of AOP may enhance the absorption of these minerals from the digestive system, thereby improving their utilization within the body. On the other hand, the study findings indicated that dietary supplementation with AOP led to a reduction in the levels of total saturated fatty acids (**SFA**) in egg yolks, specifically palmitic acid (C16:0) and behenic acid (C22:0). Under certain physiological conditions and dietary factors, de novo lipogenesis (**DNL**) could be significantly stimulated, leading to elevated tissue levels of palmitic acid and disruption of the regulatory control over its tissue concentration (Wilke et al., 2009). Similarly, chickens supplemented with antioxidants derived from *flaxseed, Rhus coriaria* seed, or *Zingiber officinale* root powder exhibited a reduction in the saturated fatty acid content of their egg yolks (Gurbuz and Salih, 2017). The AOP intervention collectively improved both the external and internal quality of the eggs, thereby enhancing their suitability for further production or sale.

The ovary played a crucial role in determining a female's egg-laying capacity, and by enhancing ovarian function, it improved the ability of laying hens to produce eggs. The diet supplemented with AOP positively impacted the oviduct weight, oviduct index, number of segmented yellow follicles, and number of preovulatory follicles, as evidenced by this study. On the other hand, the reproductive system's layers experienced a complex series of biochemical and physiological changes alongside the formation and maturation of ovarian follicles. Hormones primarily induced follicle formation (Yang et al., 2018). The AOP group was found to have higher levels of serum FSH and E2 in comparison to the control group, according to the study. It was widely recognized that gonadotropins, particularly the pituitary gland's secreted LH and FSH, were vital in stimulating oocyte growth and follicular cell proliferation, essential processes for follicle formation (Oduwole et al., 2021). Moreover, in the ovary, the AOP-supplemented diet down-regulated INH expression while up-regulating FSHR and GnRH expression. According to reports, FSH could increase the weight and volume of follicles while decreasing the number of atresia small follicles (Ma et al., 2020). According to earlier studies, the high polyphenol content of *F. velutipes* stem waste raised serum hormone levels as well as the expression of genes and proteins linked to the follicle-stimulating hormone receptor (**FSHR**) and luteinizing hormone receptor (**LHR**) signal pathways (Sun et al., 2023). Taken together, these findings provide compelling evidence for the beneficial impact of AOP on the egg production capacity of laying breeder hens.

The laying process was closely associated with heightened susceptibility to inflammation and oxidative stress. Antioxidant enzymes, such as GSH-Px, CAT, and SOD, were pivotal in defending the organism against oxidative damage, which results from the production of reactive oxygen species (**ROS**) and the antioxidant defense system being out of balance (Mishra and Jha, 2019)**.** Malondialdehyde (**MDA**), a byproduct of lipid peroxidation initiated by free radicals, serves as a marker to determine the level of oxidative stress, while T-AOC reflects the comprehensive antioxidant capacity (Cao et al., 2016; Liu et al., 2021). The findings from this study indicate that the supplementation of AOP led to an increase in the activity of T-AOC and SOD, alongside a decrease in the content of MDA in the serum. In a study by (Bayati and Yazdanparast, 2011), yakuchinone B, a component of *Alpinia oxyphylla* seeds, was demonstrated to reduce levels of ROS and inhibit the production of lipofuscin, thus mitigating cellular aging. In this study, GPx gene expression was significantly upregulated in the liver and jejunum upon dietary addition of 3 g/kg of AOP. Additionally, the expression of GPx in the ovary, CAT in the jejunum, and SOD1 in both the ovary and the jejunum was upregulated. (Liu et al., 2023) reported that extracts from *Lonicera floss* and *Cnicus japonicus* could improve egg quality by affecting the production of cytokines associated with inflammation, antioxidant status, and eggshell matrix proteins in the oviduct of laying hens. Additionally, the Nrf2 was widely recognized for its essential role in regulating antioxidant genes and coordinating various cellular defense mechanisms against oxidative stress (Liu et al., 2018). The activation of the Nrf2/HO-1 pathway reduced oxidative stress in tissues by increasing the expression of several antioxidant genes (Sahin et al., 2014). Consistent with these results, the present study showed that AOP supplementation up-regulated Nrf2 gene mRNA expression in the ovary. Specifically, at a dosage of 3 g/kg, AOP elevated the expression of HO-1 in both the ovaries and jejunum. Moreover, (Wang et al., 2013) reported that *A. oxyphylla* exhibited antioxidant activity, primarily attributed to its total phenolic components. Therefore, the antioxidant function of AOP may be the cause of the laying breeder hens' elevated antioxidant levels.

Cell apoptosis brought on by reproductive tract infections was one of the most frequent causes of reproductive dysfunction in laying hens (Li et al., 2016). Apoptosis, a regulated process of cell death, played crucial roles in development, maintaining balance, and eliminating damaged cells and tissues (Armstrong, 2007). Studies indicated that ovarian-cell apoptosis was more prevalent in breeder hens that produce fewer eggs (Wang et al., 2021). Bax exhibits pro-apoptotic activity, while Bcl-2 was the most prominent anti-apoptotic protein within the Bcl-2 family, serving as a crucial regulator of cellular death (Birkinshaw and Czabotar, 2017). Upon receiving an apoptotic signal, apoptotic initiators were activated, followed by a downstream apoptotic executor, such as caspase 3, which resulted in apoptosis (Boice and Bouchier-Hayes, 2020). The results of this experiment partially explained the process of apoptosis. The results showed that dietary AOP supplemented with 3 g/kg decreased the concentration of Caspase 8 in serum, decreased the expression of Bax and Caspase3, and increased the ratio of Bcl-2/Bax in ovary. Research indicated that *Alpiniae oxyphyllae Fructus* (**AOF**) decreased H_2_O_2_-induced damage to PC12 cells by upregulating Bcl-2 expression and downregulating Bax and caspase-3 expression in PC12 cells (Li et al., 2022). Based on these findings, it's plausible that AOP might modulate apoptosis in laying breeder hens.

Enhancing immunity was vital for shielding the body from parasite infections, cancer, viral infections, and other diseases (Yang et al., 2020). Serum immunoglobulin levels served as markers of the host's overall humoral immune function, and immunoglobulins such as IgG, IgM, and IgA were pivotal in safeguarding the host against infections and other harmful microorganisms (Lu et al., 2019). Our study results suggested that supplementing laying hens' diets with AOP could enhance their immunological well-being, as evidenced by increased levels of serum IgA and IgM.

T helper type 1 cytokines (e.g., IL-2, TNF-α, and IFN) were responsible for cell-mediated immune responses, while T helper type 2 cytokines (e.g., IL-4, IL-6, and IL-10) promoted B-cell growth, differentiation, and humoral immune responses (Kohut et al., 2002). Our study found an increase in IL-4 expression in the liver, a decrease in mRNA levels of IL-1β expression in the liver, IL-1β and IL-6 expression in the ovary, and NLRP3 in the jejunal mucosa. Additionally, there was a significant rise in the mRNA level of IL-10 in the liver and ovaries with dietary AOP at 3 g/kg. These findings suggested potent anti-inflammatory properties of AOP. Our findings align with a recent study demonstrating that the polysaccharides from *A. oxyphyllae fructus* elevated TNF-α, IL-6, IL-10, and TGF-β production, leading to the activation of RAW264.7 macrophages and enhancement of the Th2-type immune response (Yang et al., 2021). Our findings prompted speculation that AOP might modulate the immune systems of laying breeder hens.

Intestinal morphology, including villus height, crypt depth, and intestinal wall thickness, along with the V/C ratio, served as crucial indicators of intestinal health in animals as they directly impact the absorptive capacity of the intestinal mucosa (Wu et al., 2018). A greater VCR fosters an environment in the gut that was favorable to effective nutrition absorption and digestion (Montagne et al., 2003). In our observation of the jejunum, we found that AOP supplementation in the diet decreased crypt depth and augmented the VCR. Additionally, the AOP group displayed a markedly higher VH/CD ratio compared to the CON group. Plant-derived feed additives may improve the efficiency of nutrient absorption by increasing the ratio of villus height to crypt depth in the small intestine (Khattak et al., 2014). There was evidence that oregano essential oil improved intestinal nutrient absorption by increasing villus height and reducing crypt depth (Amer et al., 2021). We found that hens fed with 1 g/kg AOP enhanced the levels of mRNA for PEPT1 and SGLT1 in jejunal mucosa, which suggested that this additive might promote the absorption of nutrients. Tight junctions, comprising transmembrane proteins such as CLDN1, Occludin, and ZO1, served as critical physical barriers in maintaining intestinal barrier integrity. These proteins played a crucial role in regulating the permeability of the intestinal epithelium (Gong et al., 2020; Amevor et al., 2022). Additionally, mucin-2 has been demonstrated to be vital for preserving the integrity of the intestinal mucosal barrier, safeguarding the intestinal epithelium, and preventing infections (Mcguckin et al., 2011). When AOP was added to the diet at a concentration of 1 g/kg, there was a notable increase in the VCR and a decrease in crypt depth in the jejunum. These alterations correlated with elevated mRNA levels of MUC-2 and occludin in the jejunal mucosa. Prior studies investigating ducks administered with 30 or 130 mg/kg AOE have similarly demonstrated positive alterations in jejunal architecture, aligning with our observations and implying potential protective advantages for gut health and small intestine integrity (Ji et al., 2021). Overall, these findings provided support for the hypothesis that dietary AOP might enhance the physical barrier function of laying breeder hens.

It was well-established that the gut microbiota had a significant impact on the host's gastrointestinal system development, thereby exerting a substantial impact on immunological function, growth performance, energy balance, and nutrient utilization (Backhed et al., 2004; Yang et al., 2022). In our study, we examined the impact of dietary AOP on cecal microbial composition. Prior research has indicated that supplementation with 30 mg/kg of *A. oxyphylla* extract led to increased jejunal villus height, altered microbial composition, and demonstrated potential for preserving intestinal health in ducks (Ji et al., 2021). While no discernible variations were found between the AOP and CON groups regarding alpha diversity indices, the beta diversity analysis revealed a distinct separation, suggesting variations in microbial communities between the 2 groups. *Bacteroidetes, Firmicutes*, and *Proteobacteria* were found to dominate the microbial community in hens, which was consistent with the results of (Zhao et al., 2019), who found that *Bacteroidetes* and *Firmicutes* were the predominant phyla in the cecum, with a beneficial role in nutrient digestion and absorption (Mohd et al., 2015; Xiao et al., 2017). The composition of the cecal microbial community in the AOP group of hens changed as a result of our study's AOP dietary supplementation. Notably, there was an increase in the abundance of *Oscillospira, Phascolarctobacterium, Faecalibacterium, Parabacteroides, Blautia, Odoribacter*, and *Megasphaera* genera, alongside a reduction in the richness of *Clostridiaceae_Clostridium* at the genus level. *Oscillospira*, a genus of anaerobic bacteria within the *Firmicutes* phylum, includes species like *O. ruminantium*, known for its potential to produce butyrate (Gophna et al., 2016). *Phascolarctobacterium*, another butyrate-producing bacterium, was recognized for its anti-inflammatory properties and role in promoting intestinal epithelial integrity (Louis and Flint, 2009). *Faecalibacterium*, an essential butyrate producer in chicken ceca, contributed to gut health (Duncan et al., 2002). *Blautia*, identified as a functional genus with probiotic potential, has been associated with reducing inflammation, metabolic diseases, and exhibiting antimicrobial activity against specific microorganisms (van Leeuwen et al., 2020). These findings suggested that dietary supplementation with AOP might enhance intestinal barrier function by promoting the colonization of beneficial bacteria.

We conducted a Spearman's correlation analysis to investigate the relationship between the observed changes in intestinal architecture and cecal microbial composition. Our findings revealed correlations between the genera *Oscillospira, Blautia*, and *Megasphaera* with indicators of intestinal integrity and laying performance. Previous research has indicated the potential of *Oscillospira* to produce short-chain fatty acids (**SCFA**) like butyrate, positioning it as a promising candidate for future probiotic applications (Yang et al., 2021). *Blautia*, a type of probiotic bacteria, contributed to both the metabolism of carbohydrates and the synthesis of fatty acids (Liu et al., 2021). *Megasphaera*, known for its production of short-chain fatty acids (**SCFA**), essential amino acids, and vitamins, could adjust the host's immune response to favorably impact host health (Shetty et al., 2013; Carey et al., 2021). SCFAs, which were produced through bacterial fermentation, played crucial roles in maintaining gut integrity, regulating the immune system, reducing inflammation, and modulating epithelial gene expression (Parada et al., 2019). Therefore, it was plausible to suggest that supplementing the diet with AOP might enhance laying efficiency and promote gut health through a rise in the number of good bacteria and fostering favorable bacterial interactions.

## CONCLUSIONS

In conclusion, our research indicated that adding AOP to the diet improved the productivity of laying breeder hens. The observed beneficial effects of AOP on broiler chickens were likely attributable to improvements in immunity, intestinal health, antioxidant capacity, and reproductive hormone levels. These findings suggested that AOP has potential as a chicken-produced antibiotic substitute.

## Appendix Supplementary materials

Image, application 1

## References

[bib0001] Ali A. (2015). Flavonoids: health promoting phytochemicals for animal production-a review. J. Anim. Health Prod..

[bib0002] Al-Khalaifa H., Al-Nasser A., Al-Surayee T., Al-Kandari S., Al-Enzi N., Al-Sharrah T., Ragheb G., Al-Qalaf S., Mohammed A. (2019). Effect of dietary probiotics and prebiotics on the performance of broiler chickens. Poult. Sci..

[bib0003] Amer S.A., Tolba S.A., AlSadek D., Abdel F.D., Hassan A.M., Metwally A.E. (2021). Effect of supplemental glycerol monolaurate and oregano essential oil blend on the growth performance, intestinal morphology, and amino acid digestibility of broiler chickens. BMC Vet. Res..

[bib0004] Amevor F.K., Cui Z., Du X., Ning Z., Deng X., Xu D., Shu G., Wu Y., Cao X., Shuo W., Tian Y., Li D., Wang Y., Zhang Y., Du X., Zhu Q., Han X., Zhao X. (2022). Supplementation of dietary quercetin and vitamin e promotes the intestinal structure and immune barrier integrity in aged breeder hens. Front. Immunol..

[bib0005] Aoac I. (2010).

[bib0006] Armstrong J. (2007). Mitochondrial medicine: pharmacological targeting of mitochondria in disease. Br. J. Pharmacol..

[bib0007] Backhed F., Ding H., Wang T., Hooper L.V., Koh G.Y., Nagy A., Semenkovich C.F., Gordon J.I. (2004). The gut microbiota as an environmental factor that regulates fat storage. Proc. Natl. Acad. Sci. U S A.

[bib0008] Bayati S., Yazdanparast R. (2011). Antioxidant and free radical scavenging potential of yakuchinone B derivatives in reduction of lipofuscin formation using H2O2-treated neuroblastoma cells. Iran Biomed. J..

[bib0009] Bello A., Dersjant-Li Y., Korver D.R. (2020). Effects of dietary calcium and available phosphorus levels and phytase supplementation on performance, bone mineral density, and serum biochemical bone markers in aged white egg-laying hens. Poult. Sci..

[bib0010] Birkinshaw R.W., Czabotar P.E. (2017). The BCL-2 family of proteins and mitochondrial outer membrane permeabilisation. Semin. Cell Dev. Biol..

[bib0011] Boice A., Bouchier-Hayes L. (2020). Targeting apoptotic caspases in cancer. Biochimica et Biophysica Acta (BBA) – Mol. Cell Res..

[bib0012] Cao W., Xiao L., Liu G., Fang T., Wu X., Jia G., Zhao H., Chen X., Wu C., Cai J., Wang J. (2016). Dietary arginine and N-carbamylglutamate supplementation enhances the antioxidant statuses of the liver and plasma against oxidative stress in rats. Food Funct..

[bib0013] Carey M.A., Medlock G.L., Alam M., Kabir M., Uddin M.J., Nayak U., Papin J., Faruque A., Haque R., Petri W.A., Gilchrist C.A. (2021). Megasphaera in the stool microbiota is negatively associated with diarrheal cryptosporidiosis. Clin. Infect. Dis..

[bib0014] Chen F., Li H.L., Li Y.H., Tan Y.F., Zhang J.Q. (2013). Quantitative analysis of the major constituents in Chinese medicinal preparation SuoQuan formulae by ultra fast high performance liquid chromatography/quadrupole tandem mass spectrometry. Chem. Cent. J..

[bib0015] Ding S., Jiang H., Fang J. (2018). Regulation of immune function by polyphenols. J. Immunol. Res..

[bib0016] Duncan S., Hold G., Harmsen H., Stewart C., Flint H. (2002). Growth requirements and fermentation products of Fusobacterium prausnitzii, and a proposal to reclassify it as Faecalibacterium prausnitzii gen. nov., comb. nov. Int. J. Syst. Evol. Microbiol..

[bib0017] El-Sonbaty Y.A., Suddek G.M., Megahed N., Gameil N.M. (2019). Protocatechuic acid exhibits hepatoprotective, vasculoprotective, antioxidant and insulin-like effects in dexamethasone-induced insulin-resistant rats. Biochimie.

[bib0018] Feng J., Lu M., Wang J., Zhang H., Qiu K., Qi G., Wu S. (2021). Dietary oregano essential oil supplementation improves intestinal functions and alters gut microbiota in late-phase laying hens. J. Anim. Sci. Biotechnol..

[bib0019] Ghasemi H., Nari N. (2019). Effect of supplementary betaine on growth performance, blood biochemical profile, and immune response in heat-stressed broilers fed different dietary protein levels. J. Appl. Poult. Res..

[bib0020] Gong Y., Xia W., Wen X., Lyu W., Xiao Y., Yang H., Zou X. (2020). Early inoculation with caecal fermentation broth alters small intestine morphology, gene expression of tight junction proteins in the ileum, and the caecal metabolomic profiling of broilers. J. Anim. Sci. Biotechnol..

[bib0021] Gophna U., Konikoff T., Nielsen H. (2016). Oscillospira and related bacteria – from metagenomic species to metabolic features. Environ. Microbiol..

[bib0022] Gurbuz Y., Salih Y.G. (2017). Influence of sumac (Rhus Coriaria L.) and ginger (Zingiber officinale) on egg yolk fatty acid, cholesterol and blood parameters in laying hens. J. Anim. Physiol. Anim. Nutr. (Berl).

[bib0023] Jayaprakasha G., Singh R., Sakariah K.K. (2001). Antioxidant activity of grape seed (Vitis vinifera) extracts on peroxidation models in vitro. Food Chem..

[bib0024] Ji F., Gu L., Rong G., Hu C., Sun W., Wang D., Peng W., Lin D., Liu Q., Wu H., Dai H., Zhou H., Xu T. (2021). Using extract from the stems and leaves of Yizhi (Alpiniae oxyphyllae) as feed additive increases meat quality and intestinal health in ducks. Front. Vet. Sci..

[bib0025] Khan S., Moore R.J., Stanley D., Chousalkar K.K. (2020). The gut microbiota of laying hens and its manipulation with prebiotics and probiotics to enhance gut health and food safety. Appl. Environ. Microbiol..

[bib0026] Khattak F., Ronchi A., Castelli P., Sparks N. (2014). Effects of natural blend of essential oil on growth performance, blood biochemistry, cecal morphology, and carcass quality of broiler chickens. Poult. Sci..

[bib0027] Kohut M.L., Cooper M.M., Nickolaus M.S., Russell D.R., Cunnick J.E. (2002). Exercise and psychosocial factors modulate immunity to influenza vaccine in elderly individuals. J. Gerontol. A Biol. Sci. Med. Sci..

[bib0028] Korver D.R. (2023). Review: current challenges in poultry nutrition, health, and welfare. Animal.

[bib0029] Li J.T., Zhao Y.H., Lv Y., Su X., Mei W.L., Lu Y.P., Zheng P.H., Zhang Z.L., Zhang X.X., Chen H.Q., Dai H.F., Xian J.A. (2023). Evaluating the antioxidant properties of the leaves and stems of alpinia oxyphylla in vitro and its growth-promoting, muscle composition change, and antioxidative stress function on juvenile litopenaeus vannamei. Antioxidants (Basel).

[bib0030] Li J., Du Q., Li N., Du S., Sun Z. (2021). Alpiniae oxyphyllae Fructus and Alzheimer's disease: an update and current perspective on this traditional Chinese medicine. Biomed. Pharmacother..

[bib0031] Li R., Guo K., Liu C., Wang J., Tan D., Han X., Tang C., Zhang Y., Wang J. (2016). Strong inflammatory responses and apoptosis in the oviducts of egg-laying hens caused by genotype VIId Newcastle disease virus. BMC Vet. Res..

[bib0032] Li R., Wang L., Zhang Q., Duan H., Qian D., Yang F., Xia J. (2022). Alpiniae oxyphyllae fructus possesses neuroprotective effects on H(2)O(2) stimulated PC12 cells via regulation of the PI3K/Akt signaling pathway. Front. Pharmacol..

[bib0033] Liu M., Zhou J., Li Y., Ding Y., Lian J., Dong Q., Qu Q., Lv W., Guo S. (2023). Effects of dietary polyherbal mixtures on growth performance, antioxidant capacity, immune function and jejunal health of yellow-feathered broilers. Poult. Sci..

[bib0034] Liu S.J., Wang J., He T.F., Liu H.S., Piao X.S. (2021). Effects of natural capsicum extract on growth performance, nutrient utilization, antioxidant status, immune function, and meat quality in broilers. Poult. Sci..

[bib0035] Liu X., Mao B., Gu J., Jiaying W., Cui S., Wang G., Zhao J., Zhang H., Chen W. (2021). Blautia —a new functional genus with potential probiotic properties?. Gut Microbes..

[bib0036] Liu X., Peng C., Qu X., Guo S., Chen J.F., He C., Zhou X., Zhu S. (2019). Effects of Bacillus subtilis C-3102 on production, hatching performance, egg quality, serum antioxidant capacity and immune response of laying breeders. J. Anim. Physiol. Anim. Nutr. (Berl.).

[bib0037] Liu X., Lin X., Zhang S., Guo C., Li J., Mi Y., Zhang C. (2018). Lycopene ameliorates oxidative stress in the aging chicken ovary via activation of Nrf2/HO-1 pathway. Aging (Albany NY).

[bib0038] Liu Z.P., Chao J.R., Xu P.T., Lv H.Y., Ding B.Y., Zhang Z.F., Li L.L., Guo S.S. (2023). Lonicera flos and Cnicus japonicus extracts improved egg quality partly by modulating antioxidant status, inflammatory-related cytokines and shell matrix protein expression of oviduct in laying hens. Poult. Sci..

[bib0039] Livak K.J., Schmittgen T.D. (2001). Analysis of relative gene expression data using real-time quantitative PCR and the 2(-Delta Delta C(T)) method. Methods.

[bib0040] Louis P., Flint H.J. (2009). Diversity, metabolism and microbial ecology of butyrate-producing bacteria from the human large intestine. FEMS Microbiol. Lett..

[bib0041] Lu J., Zhang X., Liu Y., Cao H., Han Q., Xie B., Fan L., Li X., Hu J., Yang G., Shi X. (2019). Effect of fermented corn-soybean meal on serum immunity, the expression of genes related to gut immunity, gut microbiota, and bacterial metabolites in grower-finisher pigs. Front. Microbiol..

[bib0042] Ma Y., Yao J., Zhou S., Mi Y., Tan X., Zhang C. (2020). Enhancing effect of FSH on follicular development through yolk formation and deposition in the low-yield laying chickens. Theriogenology.

[bib0043] McGuckin M.A., Linden S.K., Sutton P., Florin T.H. (2011). Mucin dynamics and enteric pathogens. Nat. Rev. Microbiol..

[bib0044] Mishra B., Jha R. (2019). Oxidative stress in the poultry gut: potential challenges and interventions. Front. Vet. Sci..

[bib0045] Mohd S.M., Sieo C.C., Chong C.W., Gan H.M., Ho Y.W. (2015). Deciphering chicken gut microbial dynamics based on high-throughput 16S rRNA metagenomics analyses. Gut. Pathog..

[bib0046] Montagne L., Pluske J., Hampson D. (2003). A review of interactions between dietary fibre and the intestinal mucosa, and their consequences on digestive health in young non-ruminant animals. Anim. Feed Sci. Technol..

[bib0047] Oduwole O.O., Huhtaniemi I.T., Misrahi M. (2021). The roles of luteinizing hormone, follicle-stimulating hormone and testosterone in spermatogenesis and folliculogenesis revisited. Int. J. Mol. Sci..

[bib0048] Oteiza P.I., Fraga C.G., Mills D.A., Taft D.H. (2018). Flavonoids and the gastrointestinal tract: local and systemic effects. Mol. Aspects Med..

[bib0049] Parada V.D., De la Fuente M.K., Landskron G., Gonzalez M.J., Quera R., Dijkstra G., Harmsen H., Faber K.N., Hermoso M.A. (2019). Short chain fatty acids (SCFAs)-mediated gut epithelial and immune regulation and its relevance for inflammatory bowel diseases. Front. Immunol..

[bib0050] Park Y., Jung S., Kang S., Heo B., Arancibia P., Toledo F., Drzewiecki J., Namieśnik J., Gorinstein S. (2008). Antioxidants and proteins in ethylene-treated kiwifruits. Food Chem.

[bib0051] Parks D., Tyson G., Philip H., Beiko R. (2014). STAMP: statistical analysis of taxonomic and functional profiles. Bioinformatics (Oxford, England).

[bib0052] Qi Y., Cheng X., Jing H., Yan T., Xiao F., Wu B., Bi K., Jia Y. (2019). Effect of Alpinia oxyphylla-Schisandra chinensis herb pair on inflammation and apoptosis in Alzheimer's disease mice model. J. Ethnopharmacol..

[bib0053] Sahin K., Orhan C., Tuzcu M., Sahin N., Ali S., Bahcecioglu I.H., Guler O., Ozercan I., Ilhan N., Kucuk O. (2014). Orally administered lycopene attenuates diethylnitrosamine-induced hepatocarcinogenesis in rats by modulating Nrf-2/HO-1 and Akt/mTOR pathways. Nutr Cancer.

[bib0054] Shetty S.A., Marathe N.P., Lanjekar V., Ranade D., Shouche Y.S. (2013). Comparative genome analysis of Megasphaera sp. reveals niche specialization and its potential role in the human gut. PLoS One.

[bib0055] Sun C., Wu H., Xiao H., Nguepi Tsopmejio I.S., Jin Z., Song H. (2023). Effect of dietary Flammulina velutipes (Curt.: Fr.) stem waste on ovarian follicles development in laying hens. Ital. J. Anim. Sci..

[bib0056] Tang H., Gong Y.Z., Wu C.X., Jiang J., Wang Y., Li K. (2009). Variation of meat quality traits among five genotypes of chicken. Poult. Sci..

[bib0057] van Leeuwen S.S., Te P.E., Chatziioannou A.C., Benjamins E., Haandrikman A., Dijkhuizen L. (2020). Goat milk oligosaccharides: their diversity, quantity, and functional properties in comparison to human milk oligosaccharides. J. Agric. Food Chem..

[bib0058] Wang C.Z., Yuan H.H., Bao X.L., Lan M.B. (2013). In vitro antioxidant and cytotoxic properties of ethanol extract of Alpinia oxyphylla fruits. Pharm. Biol..

[bib0059] Wang J., Zhang H., Bai S., Zeng Q., Su Z., Zhuo Y., Mao X., Yin H., Feng B., Liu J., Zhang K., Ding X. (2021). Dietary tributyrin improves reproductive performance, antioxidant capacity, and ovary function of broiler breeders. Poult. Sci..

[bib0060] Wang K., Zhu J., Shen L. (2012). A new lignan with anti-tumour activity from Polygonum perfoliatum L. Nat. Prod. Res..

[bib0061] Wang X.C., Wang X.H., Wang J., Wang H., Zhang H.J., Wu S.G., Qi G.H. (2018). Dietary tea polyphenol supplementation improved egg production performance, albumen quality, and magnum morphology of Hy-Line Brown hens during the late laying period. J Anim Sci.

[bib0062] Wang Y., Wang M., Fan K., Li T., Yan T., Wu B., Bi K., Jia Y. (2018). Protective effects of Alpinae Oxyphyllae Fructus extracts on lipopolysaccharide-induced animal model of Alzheimer's disease. J Ethnopharmacol.

[bib0063] Wilke M.S., French M.A., Goh Y.K., Ryan E.A., Jones P.J., Clandinin M.T. (2009). Synthesis of specific fatty acids contributes to VLDL-triacylglycerol composition in humans with and without type 2 diabetes. Diabetologia.

[bib0064] Wu Y., Shao Y., Song B., Zhen W., Wang Z., Guo Y., Shahid M.S., Nie W. (2018). Effects of Bacillus coagulans supplementation on the growth performance and gut health of broiler chickens with Clostridium perfringens-induced necrotic enteritis. J Anim Sci Biotechnol.

[bib0065] Xia W.G., Chen W., Abouelezz K., Ruan D., Wang S., Zhang Y.N., Fouad A.M., Li K.C., Huang X.B., Zheng C.T. (2020). The effects of dietary Se on productive and reproductive performance, tibial quality, and antioxidant capacity in laying duck breeders. Poult Sci.

[bib0066] Xiao T., Pan M., Wang Y., Huang Y., Tsunoda M., Zhang Y., Wang R., Hu W., Yang H., Li L.S., Song Y. (2023). In vitro bloodbrain barrier permeability study of four main active ingredients from Alpiniae oxyphyllae fructus. J Pharm Biomed Anal.

[bib0067] Xiao Y., Xiang Y., Zhou W., Chen J., Li K., Yang H. (2017). Microbial community mapping in intestinal tract of broiler chicken. Poult Sci.

[bib0068] Yang J.X., Chaudhry M.T., Yao J.Y., Wang S.N., Zhou B., Wang M., Han C.Y., You Y., Li Y. (2018). Effects of phyto-oestrogen quercetin on productive performance, hormones, reproductive organs and apoptotic genes in laying hens. J Anim Physiol Anim Nutr (Berl).

[bib0069] Yang J., Li Y., Wen Z., Liu W., Meng L., Huang H. (2021). Oscillospira - a candidate for the next-generation probiotics. Gut Microbes.

[bib0070] Yang L., Ma X., Yang C., Jiang S., Yang W., Jiang S. (2022). The combination of plant extracts and probiotics improved jejunal barrier and absorption capacity of weaned piglets. Agriculture.

[bib0071] Yang X., Zhou S., Li H., An J., Li C., Zhou R., Teng L., Zhu Y., Liao S., Yang Y., Chen H., Chen Y. (2021). Structural characterization of Alpiniae oxyphyllae fructus polysaccharide 2 and its activation effects on RAW264.7 macrophages. Int. Immunopharmacol..

[bib0072] Yang X., Yang Y., Chen H., Xu T., Li C., Zhou R., Gao L., Han M., He X., Chen Y. (2020). Extraction, isolation, immunoregulatory activity, and characterization of Alpiniae oxyphyllae fructus polysaccharides. Int. J. Biol. Macromol..

[bib0073] Yong Z., Zibao H., Zhi Z., Ning M., Ruiqi W., Mimi C., Xiaowen H., Lin D., Zhixuan X., Qiang L., Weiying L., Xiaopo Z. (2022). Nootkatone, a sesquiterpene ketone from alpiniae oxyphyllae fructus, ameliorates metabolic-associated fatty liver by regulating AMPK and MAPK signaling. Front. Pharmacol..

[bib0074] Yuan Z.H., Zhang K.Y., Ding X.M., Luo Y.H., Bai S.P., Zeng Q.F., Wang J.P. (2016). Effect of tea polyphenols on production performance, egg quality, and hepatic antioxidant status of laying hens in vanadium-containing diets. Poult. Sci..

[bib0075] Zhang L., Ge J., Gao F., Yang M., Li H., Xia F., Bai H., Piao X., Sun Z., Shi L. (2023). Rosemary extract improves egg quality by altering gut barrier function, intestinal microbiota and oviductal gene expressions in late-phase laying hens. J. Anim. Sci. Biotechnol..

[bib0076] Zhang Q.Q., Chang C., Chu Q., Wang H.H., Zhang J., Yan Z.X., Song Z.G., Geng A.L. (2023). Dietary calcium and non-phytate phosphorus levels affect the performance, serum biochemical indices, and lipid metabolism in growing pullets. Poult. Sci..

[bib0077] Zhang Q., Zheng Y., Hu X., Hu X., Lv W., Lv D., Chen J., Wu M., Song Q., Shentu J. (2018). Ethnopharmacological uses, phytochemistry, biological activities, and therapeutic applications of Alpinia oxyphylla Miquel: a review. J. Ethnopharmacol..

[bib0078] Zhao Y., Li K., Luo H., Duan L., Wei C., Wang M., Jin J., Liu S., Mehmood K., Shahzad M. (2019). Comparison of the intestinal microbial community in ducks reared differently through high-throughput sequencing. Biomed. Res. Int..

[bib0079] Zou Y., Xiang Q., Wang J., Wei H., Peng J. (2016). Effects of oregano essential oil or quercetin supplementation on body weight loss, carcass characteristics, meat quality and antioxidant status in finishing pigs under transport stress. Livest. Sci..

