# Supplementary material for: Alpiniae oxyphyllae fructus improves production performance and egg quality of laying breeder hens by regulating reproductive hormones, antioxidant function, immunity and intestinal health
Source: Poult Sci. 2024 Apr 15;103(6):103770. doi: 10.1016/j.psj.2024.103770 (PMC11063526; doi:10.1016/j.psj.2024.103770)
Supplement: Supplementary file 1 [file mmc1.docx]

Supplementary Materials


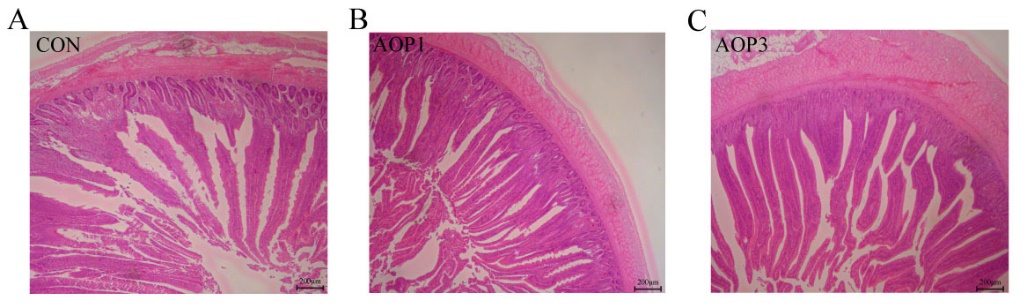


Figure S1. The jejunal tissues of laying breeder hens were stained with H&E. Scale bar: 200 μm. (A) CON = Control group (basal diet + 0 g/kg AOP); (B) AOP1 = *Alpiniae oxyphyllae Fructus* powder group 1 (basal diet + 1g/kg AOP); (C) AOP3 = *Alpiniae oxyphyllae Fructus* powder group 3 (basal diet + 3 g/kg AOP).

Table S1. The ingredient and nutrient composition of the basal diet (% as fed basis)

| Ingredients (%) | Content |
| --- | --- |
| Corn | 57.80 |
| Soybean meal | 23.90 |
| Fish meal | 3.40 |
| Soybean oil | 1.60 |
| Limestone | 6.85 |
| Gypsum Powder | 0.65 |
| Calcium hydrogen phosphate | 1.20 |
| Uniform chaff | 3.60 |
| Premixes^1)^ | 1.00 |
| Total | 100 |
| Nutrient composition^2)^ |  |
| Digestible energy (MJ/kg) | 11.31 |
| Crude protein (%) | 17.03 |
| Calcium (%) | 3.14 |
| Available phosphorus (%) | 0.36 |
| SID-Lys (%) | 0.94 |
| SID-Met (%) | 0.41 |
| SID-Cys (%) | 0.26 |

^1)^ The premix per kilogram of feed contains vitamin A 16500 IU, vitamin D 36250 IU, vitamin E 75IU, vitamin K_3_ 10 mg, vitamin B_1_ 5 mg, vitamin B_2_ 15 mg, vitamin B_6_ 15 mg, vitamin B_12_ 0.05 mg, vitamin C 186 mg, folic acid 2.5 mg, D-biotin 0.375 ng, nicotinamide 100 mg, DL-tocopheryl acetate 40 mg, Fe 200 mg, Cu 16.66 mg, Mn 184 mg, Zn 150 mg, I 0.834 mg, Se 0.416 mg, choline chloride 0.75 g, DL-methionine 1.188 g, DL-lysine 0.591 g, NaHCO_3_ 1.485 g, NaCl 2.39 g, phytase 1500 IU, xylanase 1500 IU, cellulase 250 IU, acid protease 125 IU, Amylase 25000 IU, β-mannanase 4500 IU, β-glucanase 1500 IU.

^2)^ The nutrient levels were calculated from data provided by Feed Database in China.

^3)^ Lys = lysine, Met = methionine, Cys = cysteine.

Table S2. Primers used for quantitative PCR.

| Gene | Primer sequence (5′→3′) | Accession number |
| --- | --- | --- |
| β-actin | Forward: GAGAAATTGTGCGTGACATCA | L08165.1 |
|  | Reverse: CCTGAACCTCTCATTGCCA |  |
| GAPDH | Forward: AGAACATCATCCCAGCGTCC | NM_204305 |
|  | Reverse: CGGCAGGTCAGGTCAACAAC |  |
| ESR-β | Forward: AAGAAGAGAACGCTGTGGGTAT | XM_046917703.1 |
|  | Reverse: CTCGGTGAATGGTTTGCTAGGA |  |
| FSHR | Forward: ATGTCCTTGGGTCTCACCTG | NM_205079.1 |
|  | Forward: CTGTGAAAGCTCCCTTCGGA |  |
| GnRH | Reverse: TTCACCGCATCTGTGGCAAT | NM_001080877.1 |
|  | Reverse: CTGGTAAGAGCCAGGGCATT |  |
| INH | Forward: CAAAAGGATGTGAGGAGGGTGC | NM_001031257.2 |
|  | Reverse: CCGAGGGCTGGAAGAGGTAAGT |  |
| SOD1 | Forward: ATTACCGGCTTGTCTGATGG | NM_205064.155 |
|  | Reverse: CCTCCCTTTGCAGTCACATT |  |
| GPx | Forward: AAGTGCTGCTGGTGGTCAACG | NM_001277853.2 |
|  | Reverse: GTTCTCCTGGTGCCCGAATTGG |  |
| CAT | Forward: AGCAGGTGCCTTTGGCTATT | NM_001031215.2 |
|  | Reverse: CGAGGGTCACGAACTGTATCA |  |
| Nrf2 | Forward: CGCTTTCTTCAGGGGTAGCA | NM_205117.1 |
|  | Reverse: AGTTCGGTGCAGAAGAGGTG |  |
| HO-1 | Forward: ACGAGTTCAAGCTGGTCACG | NM_205344.1 |
|  | Reverse: GGATGCTTCTTGCCAACGAC |  |
| NQO1 | Forward: GGCAATGGCAGCAGCAG | NM_001277621.1 |
|  | Reverse: AAGCACTCGGGGTTCTTGAG |  |
| IL-1β | Forward: CAGCCTCAGCGAAGAGACCTT | NM_204524.2 |
|  | Reverse: ACTGTGGTGTGCTCAGAATCC |  |
| IL-4 | Forward: TTGTTTGGGAGAGCCAGCAC | NM_001007079.1 |
|  | Reverse: GACATGGTGCCTTGAGGGAG |  |
| IL-6 | Forward: AAATCCCTCCTCGCCAATCT | HM179640 |
|  | Reverse: CCCTCACGGTCTTCTCCATAAA |  |
| IL-10 | Forward: CGCTGTCACCGCTTCTTCA | AJ621614 |
|  | Reverse: TCCCGTTCTCATCCATCTTCTC |  |
| NLRP3 | Forward: CTCAGAGGGCCTTGTGACAGTAA | NM_001001472.2 |
|  | Reverse: GGCAGATGTGAACAAGGTGA |  |
| Bax | Forward: GGTGACAGGGATCGTCACAG | XM_422067 |
|  | Reverse: TAGGCCAGGAACAGGGTGAAG |  |
| Bcl-2 | Forward: GCTGCTTTACTCTTGGGGGT | NM_205339.2 |
|  | Reverse: CTTCAGCACTATCTCGCGGT |  |
| Caspase 3 | Forward: ACTCTGGAAATTCTGCCTGATGACA | NM_204725.2 |
|  | Reverse: CATCTGCATCCGTGCCTGA |  |
| Caspase 8 | Forward: CATTGCATGGGCTGCTTAAA | NM_204592.4 |
|  | Reverse: CACCTCCCCTACGCCTATCT |  |
| p53 | Forward: CGCCGTGGCCGTCTATAAG | NM_205264.1 |
|  | Reverse: GTACAGTCAGAGCCCACCTCG |  |
| Claudin-1 | Forward: TGGAGGATGACCAGGTGAAGA | NM_001013611.2 |
|  | Reverse: CGAGCCACTCTGTTGCCATA |  |
| Occludin | Forward: ATCAACGACCGCCTCAATCAG | NM_205128.1 |
|  | Reverse: TCCTCTGCCACATCCTGGTATT |  |
| ZO-1 | Forward: CCACCTCAGAATAAGCCAGCAAT | XM_015278981.2 |
|  | Reverse: CGGTTGTAAGGAGTGACTGTT |  |
| Mucin 2 | Forward: AGGAATGGGCTGCAAGAGAC | XM_001234581.3 |
|  | Reverse: GTGACATCAGGGCACACAGA |  |
| GLUT2 | Forward: CCGCAGAAGGTGATAGAAGC | NM_205129.1 |
|  | Reverse: ATTGTCCCTGGAGGTGTT |  |
| PePT1 | Forward: TCACTGTTGGCATGTTCCT | NM_204365.2 |
|  | Reverse: TTCGCATTGCTATCACCTA |  |
| SGLT1 | Forward: TGTCTCTCTGGCAAGAACATGTC | NM_001293240.1 |
|  | Reverse: GGGCAAGAGCTTCAGGTATCC |  |
| y+LAT1 | Forward: CAGAAAACCTCAGAGCTCCCTTT | XM_040665181.1 |
|  | Reverse: TGAGTACAGAGCCAGCGCAAT |  |

GAPDH, Glyceraldehyde-3-phosphate dehydrogenase; ESR-β,Estrogen receptor beta; FSHR, Follicle-Stimulating Hormone Receptor; GnRH, Gonadotropin-releasing hormone; INH, Inhibit 1; SOD1, Superoxide Dismutase 1; GPx, Glutathione Peroxidase; CAT, Catalase; Nrf2, Nuclear factor erythroid2-related factor 2; HO-1, Heme oxygenase 1; NQO1; NAD(P)H quinone oxidoreductase 1; IL-1β, Interleukin-1β; IL-4, Interleukin-4; IL-6, Interleukin-6; IL-10, Interleukin-10; NLRP3, NOD-like receptor protein 3; Bax, Bcl2-associated X; Bcl-2, B-cell lymphoma-2; ZO-1, Zonula occludens-1; GLUT2, Glucose transporter 2; PePT1, Peptide-transporter 1; SGLT1, Sodium glucose cotransporter 1; y+LAT1, Y(+)L-Type Amino Acid Transporter 1.
